# Accumulation of prosaposin and progranulin around the subfornical organ induces polydipsia in SAP-D-deficient mice

**DOI:** 10.1016/j.bbrep.2025.102388

**Published:** 2025-12-02

**Authors:** Harumi Hisaki, Takao Susa, Noriyuki Okudaira, Miho Akimoto, Masayoshi Iizuka, Junko Matsuda, Shunya Uchida, Hiroko Okinaga, Tomoki Okazaki, Mimi Tamamori-Adachi

**Affiliations:** a Department of Biochemistry, Teikyo University School of Medicine, Itabashi, Tokyo, Japan; b Medical Education Center, Teikyo University School of Medicine, Itabashi, Tokyo, Japan; c Department of Pathophysiology and Metabolism, Kawasaki Medical School, Okayama, Japan; d Teikyo Heisei University, Toshima, Tokyo, Japan; e Department of Internal Medicine, Teikyo University School of Medicine, Itabashi, Tokyo, Japan

**Keywords:** Saposin-D, Primary polydipsia, Subfornical organ, Microglia, PSAP, PGRN

## Abstract

Prosaposin (PSAP), a precursor of saposins, is essential for lysosomal hydrolysis of sphingolipids. It binds with progranulin (PGRN) and transports from the Golgi to lysosomes, where it is processed into saposins. PSAP is also secreted and functions on various cells, including neurons. We found that PSAP is highly expressed in the subfornical organ (SFO), a thirst center, in SAP-D-deficient (SAP-D^−/−^) mice, which develop primary polydipsia. As polyuria progresses, CD68-positive active microglia infiltrate the SFO and strongly express PSAP and PGRN. Lysosomal marker LAMP1 analysis in the SFO of mice with advanced polydipsia showed increased LAMP1 expression and decreased co-localization of PSAP and LAMP1 in microglia and neurons. This suggests that SAP-D-deficient PSAP struggles to reach lysosomes, causing intracellular accumulation. c-Fos-positive cell counts in the SFO remained significantly higher in SAP-D^−/−^ mice, reflecting altered drinking behavior. These findings imply that PSAP may drive polydipsia progression.

## Highlights

• In SAP-D^−/−^ mice, as polyuria progresses, CD68-positive microglia infiltrate the SFO and strongly express PSAP and PGRN.
• SAP-D-deficient PSAP struggles to reach lysosomes, causing intracellular accumulation.
• c-Fos-positive cell counts in the SFO remained significantly higher in SAP-D^−/−^ mice, reflecting altered drinking behavior.
• PSAP may drive polydipsia progression via c-Fos induction.

## 1 Introduction

Saposins (SAPs) are membrane lipid-interacting proteins and cofactors for the physiological degradation of glycosphingolipids in lysosomes. They enable the access of certain water-soluble exohydrolases to membrane-bound glycosphingolipid and ceramide targets [1,2]. Their absence triggers diverse clinical phenotypes of sphingolipidoses, most manifested by miscellaneous neurological degenerative lesions and symptoms [[3], [4], [5], [6], [7], [8]]. Among SAPs A-D, SAP-D is proposed to be involved in ceramide hydrolysis by acid ceramidase, although the underlying molecular details have not been well characterized [8,9].

Prosaposin (PSAP) has been reported to accumulate in the brain of SAP-D deficient (SAP-D^−/−^) mice, despite the lack of changes at RNA level [10]. PSAP, a highly conserved glycoprotein and precursor of SAPs A-D [1], plays roles both intracellularly, as a regulator of lysosomal enzyme function, and extracellularly, as a secreted factor with neuroprotective and glioprotective effects under cellular stress [11]. Experimental models of neuronal injury resulted in increased PSAP levels in neurons [[11], [12], [13]]; further, increased or decreased PSAP levels have been implicated in the development of many neurodegenerative diseases [[14], [15], [16], [17], [18]].

Meanwhile, recent studies have highlighted biochemical interactions between PSAP and progranulin (PGRN) [[19], [20], [21], [22]]. PSAP and PGRN require each other to regulate lysosomal enzyme function and for the efficient migration, translocation, and transport of intracellular substances, including various intracellular metabolites involved in immune responses and carcinogenesis [[22], [23], [24], [25], [26]]. In these processes, PSAP is hydrolyzed into four SAPs and PGRN into seven granulins (GRNs). In the brain, PGRN and PSAP have been demonstrated to regulate neuroinflammation [[27], [28], [29]]. Both intracellular and extracellular PSAP and PGRN proteins levels influence each other [19,30].

We previously reported that SAP-D^−/−^ mice develop unique primary polydipsia and subsequent polyuria [31]. In those mice, histologically infiltrative inflammatory changes were observed around the subfornical organ (SFO), the thirst center in the cerebrum located near the third ventricle; however, the underlying pathophysiological consequences remained largely elusive.

Thirst is the basic instinct to drink water. The SFO is one of several circumventricular organs (CVO) nuclei activated by thirst-inducing stimuli such as water deprivation [32]. This nucleus lacks the blood-brain barrier due to the presence of fenestrated capillaries, and has been suggested to function as an osmolality sensor in the brain [[32], [33], [34]]. Oka et al. reported that nuclear expression of the immediate early gene c-Fos, a molecular marker of neuronal activity, in neurons in SFO strongly correlated with drinking behavior, and that c-Fos induction in those cells, i.e., the activation, is trigger water-drinking responses [[35], [36], [37], [38], [39], [40], [41], [42], [43], [44]].

In this study, we investigated PSAP and PGRN expression at the cell and intracellular level, around the SFO in SAP-D^−/−^ mice, and the involvement of those proteins in the development of polydipsia in SAP-D^−/−^, with respect to c-Fos expression in that area. As previously reported [31], polydipsia symptoms emerge mildly at 3 months, intensify progressively, peak reproducibly at 10 months (Supplemental Fig. S1), and stabilize thereafter. Additionally, symptoms are more severe in females compared with males. Therefore, unless otherwise specified, 10-month-old female mice were used in experiments.

## 2 Results

### 2.1 PSAP and PGRN protein expression

**Fig. 1:**
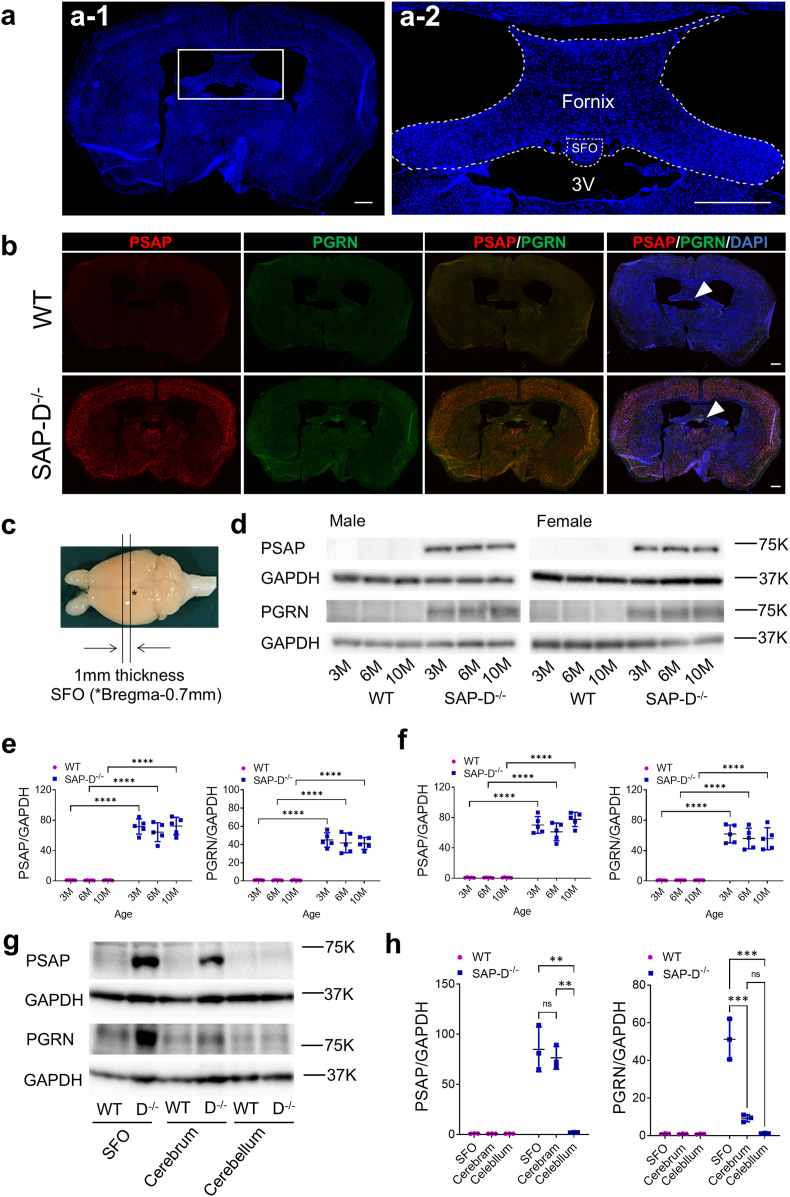
Increased protein levels of PSAP and PGRN within the SFO of SAP-D^−/−^ mice **a**–**b)**, Coronal brain section at 0.7–0.8 mm posterior to bregma containing the SFO. a: DAPI staining. a-2: Enlarged view of the white square in a-1. SFO: subfornical organ, 3V: third ventricle. b; Double immunofluorescent staining of PSAP (red) and PGRN (green) in 10-month-old female WT and SAP-D^−/−^ mice. DAPI (blue) staining showed the nuclei (**a** and **b**). Scale bar, 500 μm (**a** and **b**). White arrowheads indicate the SFO regions (**b**). **c)** Cerebral region from 3-, 6-, and 10-month-old male and female mice containing the SFO (0.7–0.8 mm posterior to ∗bregma) used for protein extraction and Western blot (**d**–**h**) using anti- PSAP, PGRN, and GAPDH antibodies. Quantification normalized to GAPDH expression and represented as the mean ± SD of three mice for each group.  and  indicate the individual values in each group (**e**, **f**, **h**). **d**–**f)** PSAP and PGRN protein levels in the SFO were remarkably increased. Their quantification by densitometric analysis is represented in **e** for male and **f** for female, respectively. **e**) For PSAP/GAPDH, two-way ANOVA revealed a significant main effects of genotype (F(1,24) = 547.7, *p* < 0.0001, ηp^2^ = 0.48, 95 % CI [−74.51, −62.43]), with no effect of age (*p* = 0.46) or genotype × age interaction (*p* = 0.47). Post-hoc Tukey's tests showed that SAP-D^−/−^ differed from WT at 3 M (*p* < 0.0001, Cohen's *d* = 9.99, 95 % CI [−86.30, −54.96]), 6 M (*p* < 0.0001, Cohen's *d* = 7.24, 95 % CI [−79.04, −47.70]), and 10 M (*p* < 0.0001, Cohen's *d* = 8.77, 95 % CI [−87.09, −55.75]). For PGRN/GAPDH, two-way ANOVA revealed a significant main effect of genotype (F(1,24) = 354.2, *p* < 0.0001, ηp^2^ = 0.48, 95 % CI [−46.39, −37.22]), with no effect of age (*p* = 0.73) or genotype × age interaction (*p* = 0.76). Post-hoc Tukey's tests showed that SAP-D^−/−^ differed from WT at 3 M (*p* < 0.0001, Cohen's *d* = 7.87, 95 % CI [−52.71, −28.91]), 6 M (*p* < 0.0001, Cohen's *d* = 5.35, 95 % CI [−52.82, −29.02]), and 10 M (*p* < 0.0001, Cohen's *d* = 8.77, 95 % CI [−52.32, −28.52]). **f**) For PSAP/GAPDH, two-way ANOVA revealed a significant main effect of genotype (F(1,24) = 611.1, *p* < 0.0001, ηp^2^ = 0.48, 95 % CI [−74.42, −62.95]), with no effect of age (*p* = 0.07) or genotype × age interaction (p = 0.07). Post-hoc Tukey's tests showed that SAP-D^−/−^ differed from WT at 3 M (*p* < 0.0001, Cohen's *d* = 9.99, 95 % CI [−86.30, −54.96]), 6 M (*p* < 0.0001, Cohen's *d* = 7.24, 95 % CI [−79.04, −47.70]), and 10 M (*p* < 0.0001, Cohen's *d* = 8.77, 95 % CI [−87.09, −55.75]). For PGRN/GAPDH, two-way ANOVA revealed a significant main effect of genotype (F(1,24) = 273.5, *p* < 0.0001, ηp^2^ = 0.47, 95 % CI [−63.96, −49.76]), with no effect of age (*p* = 0.71) or genotype × age interaction (p = 0.70). Post-hoc Tukey's tests showed that SAP-D^−/−^ differed from WT at 3 M (*p* < 0.0001, Cohen's *d* = 7.31, 95 % CI [−73.43, −36.60]), 6 M (*p* < 0.0001, Cohen's *d* = 5.71, 95 % CI [−73.04, −36.22]), and 10 M (*p* < 0.0001, Cohen's *d* = 5.35, 95 % CI [−73.08, −36.26]). **g**–**h**) Comparison of PSAP and PGRN protein expression in the SFO, whole cerebrum, and cerebellum. The quantitative analysis is shown in **h**. **h**) For PSAP/GAPDH, one-way ANOVA revealed a significant effect in SAP-D^−/−^ mice (F(2,6) = 30.06, *p* = 0.0007, η^2^ = 0.90), but not in WT mice (*p* = 0.3461). Tukey's post hoc tests showed significant differences for SFO versus cerebellum (*p* = 0.0010, Cohen's *d* = 5.26, 95 % CI [46.80, 119.1]) as well as and cerebrum versus cerebellum (*p* = 0.0018, Cohen's *d* = 9.34, 95 % CI [38.39, 110.6]). There was no significant difference for SFO versus cerebrum (*p* = 0.76, Cohen's *d* = 0.47, 95 % CI [−27.71, 44.54]). For PGRN/GAPDH, one-way ANOVA revealed a significant effect in SAP-D^−/−^ mice (F(2,6) = 54.42, *p* = 0.0001, η^2^ = 0.94), but not in WT mice (*p* = 0.6327). Tukey's post hoc tests showed significant differences for SFO versus cerebrum (*p* = 0.0005, Cohen's *d* = 5.43, 95 % CI [26.19, 57.84]) as well as and SFO versus cerebellum (*p* = 0.0005, Cohen's *d* = 5.43, 95 % CI [26.19, 57.84]), but not for cerebrum versus cerebellum (*p* = 0.3274, Cohen's *d* = 6.06, 95 % CI [−7.72, 23.92]). ns: no significant difference. ∗∗∗∗*p* < 0.0001. ∗∗∗*p* < 0.001. ∗∗*p* < 0.01.

To investigate the distribution of PSAP and PGRN proteins around the SFO (Fig. 1a), we performed immunofluorescent staining of coronal sections containing the SFO. As shown in Fig. 1b, higher PSAP expression was observed throughout the brain, including the cerebral cortex and the SFO, in SAP-D^−/−^ mice, whereas PGRN accumulated only in the periphery of the SFO. Both PSAP and PGRN were more strongly expressed in SAP-D^−/−^ than in WT mice. Fig. 1d shows the Western blot results of PSAP and PGRN in samples from the SFO, the slices of brain containing the cerebral thirst center, SFO and medial basal hypothalamus, as illustrated in Fig. 1c. There was a significant difference in the densities of both PSAP and PGRN proteins in the SFO between SAP-D^−/−^ and WT mice (Fig. 1d and Supplemental Fig. S2). For PSAP, quantitative analysis revealed a 70∼80-fold increase in the SFO region in SAP-D^−/−^ compared to that in WT, at 3, 6, and 10-months-old, in both sexes (Fig. 1d–f). For PGRN, the expression increased 40∼50-fold in SAP-D^−/−^ mice compared with WT (Fig. 1d–f). PSAP was expressed in the SFO as well as in the whole cerebrum, including the SFO region, whereas PGRN expression was observed specifically in the SFO region (Fig. 1g and h), consistent with histological analysis (Fig. 1a and b), in SAP-D^−/−^ mice. Quantitative RT-PCR analysis showed that neither PSAP nor PGRN expression changed at any time in between both SAP-D^−/−^ and WT mice, suggesting that the upregulation of PSAP and PGRN levels observed in SAP-D^−/−^ mice occurred at a post transcriptional level (Supplemental Fig. S3). In 1-month-old SAP-D^−/−^ mice, before the onset of polydipsia, the PSAP level had already increased to that at 10-month-old whereas the PGRN level was yet unchanged (Supplemental Fig. S4a–c).

### 2.2 Localization of PSAP and PGRN in the SFO

**Fig. 2:**
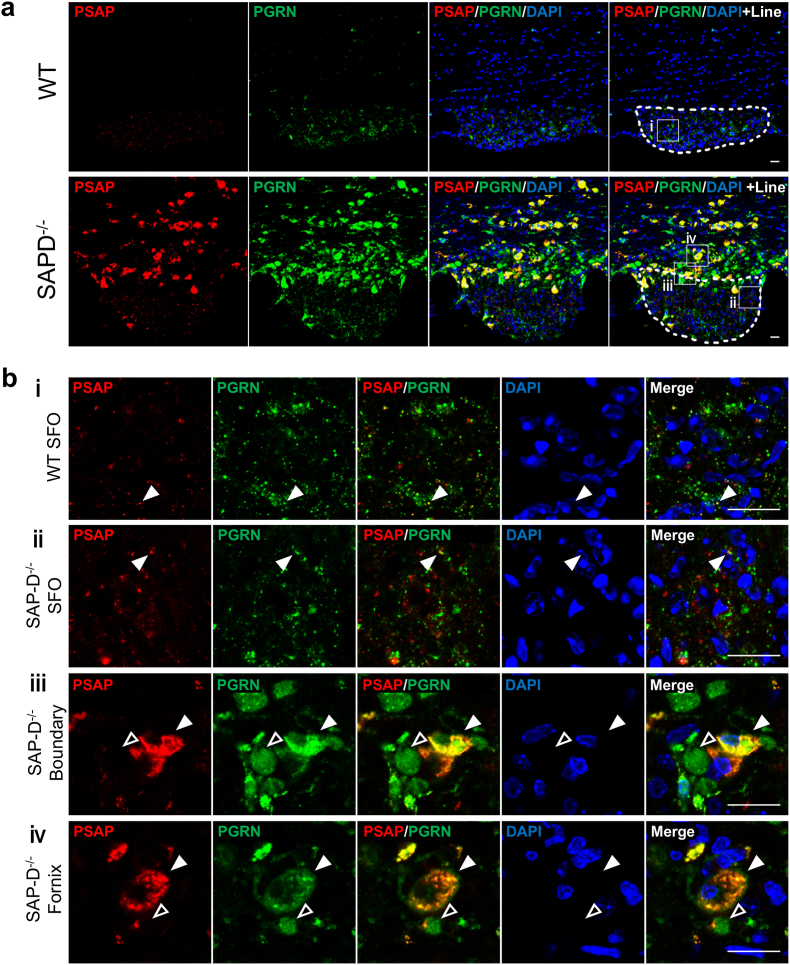
Increased PSAP and PGRN immunostaining in the SFO and its surrounding tissues in SAP-D^−/−^ mice **a)** Double immunofluorescent staining of PSAP (red) and PGRN (green) around the SFO in 10-month-old female WT and SAP-D^−/−^ mice. The white dotted lines enclose the SFO. **b**) enlarged white ⅰ-iv squares in **a,** as indicated. White arrowheads indicate co-staining with anti-PGRN and PSAP antibodies. Open arrowheads indicate staining with PGRN alone. Nuclei are labeled by DAPI (blue) staining. All scale bars, 20 μm.

To further analyze the expression of both proteins around the SFO, we performed double immunostaining for PSAP and PGRN (Fig. 2a and b). As shown in Fig. 2a, PSAP and PGRN strongly accumulated in the fornix and the boundary of the SFO in SAP-D^−/−^, but not in WT mice.

In the magnified images of the SFO (Fig. 2b, ⅰ and ⅱ), PSAP staining appeared like dots in extranuclear regions; those signals were stronger in SAP-D^−/−^ mice. The labeling for PGRN was similar to that of PSAP, but intensities did not differ between WT and SAP-D^−/−^ mice (Fig. 2b, ⅰ and ⅱ). Some co-localization of PSAP and PGRN was observed both in WT and SAP-D^−/−^ (white arrowheads) mice (Fig. 2b, ⅰ and ⅱ). In the magnified images of the fornix and the boundary, which is between SFO and fornix, of SAP-D^−/−^ mice, cells with strong PGRN and PSAP coexpression in the extranuclear region (white arrowheads) and cells with only PGRN expression (open arrowheads) were observed (Fig. 2b, ⅲ and ⅳ). Three-dimensional observations suggested that these PGRN-only cells lacked nuclei, indicating that they were not typical cell bodies (Supplemental Fig. 5a). The immunoreactive structures of PSAP and PGRN clearly differed in the SFO and the fornix/boundary. Antigen absorption experiments validated the specificity of PSAP and PGRN antibodies and confirmed that the observed immunoreactivity was specific to the antigen of interest (Supplemental Fig. S6). These immunohistological findings confirmed strong PSAP and PGRN expression around the SFO in SAP-D^−/−^ mice.

### 2.3 Cells with strong PGRN and PSAP coexpression are activated microglia/macrophages

Mendsaikhan et al. reported that PGRN immunoreactive structures in microglia are larger than those of neurons and do not localize in glial fibrillary acidic protein-positive astrocytes [14,45]. Thus, we considered that some cells expressing PGRN and/or PSAP at the boundary between SFO and fornix could be microglia. We performed co-staining with several microglia markers as well as PGRN and PSAP. CD68 is a marker for activated microglia/macrophages, and IBA-1 is expressed both in activated- and inactivated-microglia/macrophages [46,47]. Further, TMEM119 is a cell surface highly specifically expressed on microglia, but not on other immune cells such as macrophages or in the neuronal lineage [47].

**Fig. 3:**
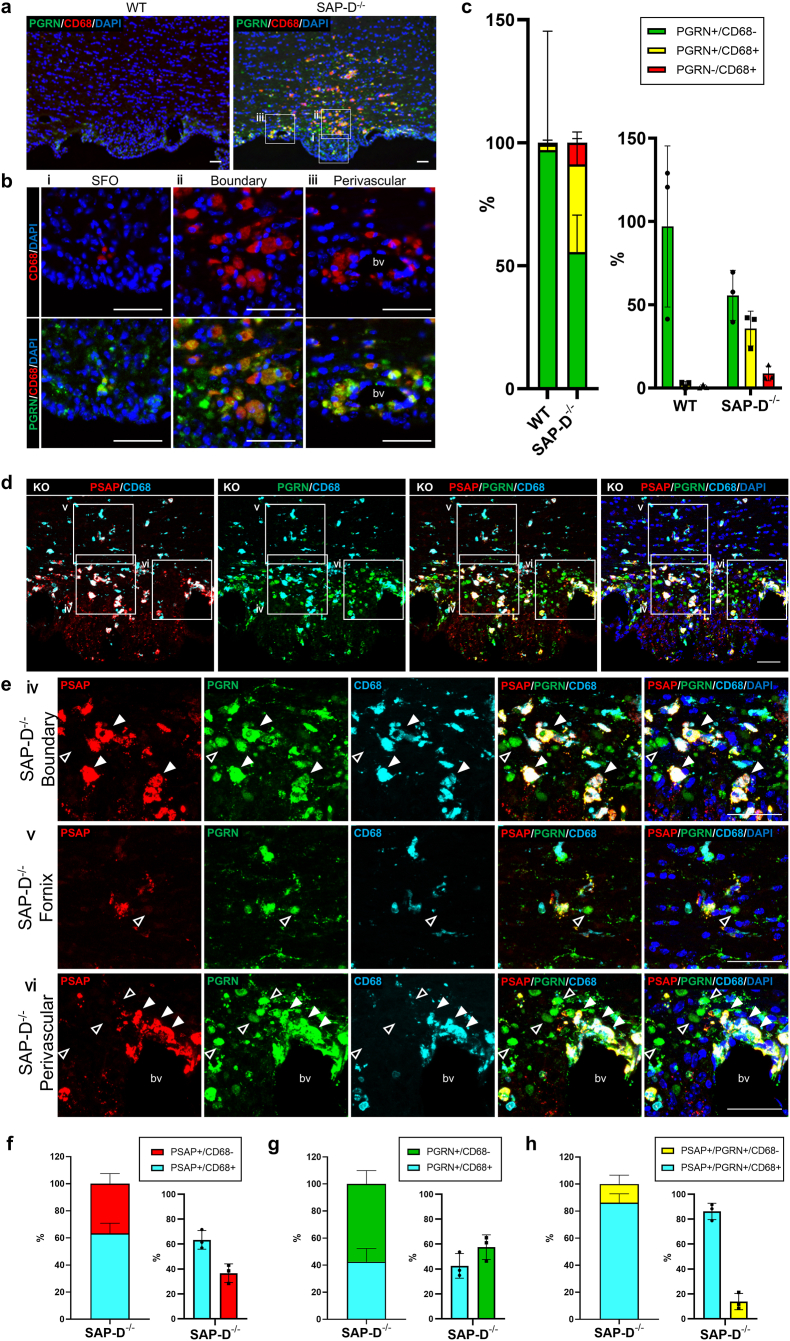
Infiltration of CD68-positive activated microglia/macrophages co-expressing PSAP and PGRN into the SFO and surrounding tissues **a**–**b)** Double immunofluorescent staining of PGRN (green) and CD68 (red) around the SFO in 10-month-old-female WT and SAP-D^−/−^ mice. **b**, Magnified images of the indicated white squares in **a**. ⅰ: SFO, ⅱ: Fornix, and ⅲ: Perivascular, bv: blood vessel. **c**) Quantification of PGRN- and/or CD68-staining in the SFO and surrounding areas in WT and SAP-D^−/−^ mice. Data are shown as the mean ± SD (n = 3). The left panel presents a stacked bar chart, whereas the right panel shows the individual data values in a bar chart format. **d**–**h**) Triple immunofluorescent staining of PSAP (red), PGRN (green), and CD68 (cyan) around the SFO in 10-month-old-female SAP-D^−/−^ mice. **e)** Magnified images of the indicated white squares in **d)** ⅳ: Boundary, ⅴ: Fornix, and ⅵ: Perivascular. White arrowheads indicate triple co-staining with PSAP, PGRN, and CD68. Open arrowheads indicate PGRN signals alone. Co-localization rates of CD68 positive areas in PSAP (**f**), PGRN (**g**), and PSAP-PGRN staining areas (**h**) around the SFO of SAP-D^−/−^ mice, respectively. **f-h**) The left panel presents a stacked bar chart, whereas the right panel presents the individual data values in a bar chart format. Data are shown as mean ± SD (n = 3). Nuclei are labeled by DAPI (blue) staining. All scale bars, 50 μm.

In SAP-D^−/−^ mice, a number of CD68-, TMEM119-, and IBA1-positive cells were found around the SFO (i.e., the boundary region between the SFO and fornix including the perivascular region; Fig. 3a, b, d and e, Supplemental Fig. S7a–d), indicating marked infiltration by microglia/macrophages into those regions. Analysis of CD68-positive microglia/macrophages at different postnatal stages revealed that “ramified”, stable microglia weakly express CD68 in 1–3-month-old SAP-D^−/−^ mice. Conversely, in 6–10-month-old SAP-D^−/−^ mice, “ameboid”, activated microglia strongly expressed CD68, and PSAP expression was observed (Supplemental Fig. S8), while few of these cells were observed in WT mice (Fig. 3a, b and Supplemental Fig. S8) [46], suggesting that, in parallel with polydipsia advance, highly-active microglia infiltration around the SFO of SAP-D^−/−^ mice is progressed. Approximately 50 % of CD68 microglia were also positive for IBA1 (Supplemental Fig. S9). Quantitative analysis showed that 80 % of CD68-positive areas co-stained with PGRN (Fig. 3c). The rate of CD68-positive areas in PGRN- and PSAP-positive areas was 43 % and 62 %, respectively (Fig. 3d–g). Triple staining with anti-PGRN, anti-PSAP, and anti-CD68 antibodies revealed that 87 % of areas stained with both PGRN and PSAP were CD68 positive (white arrowheads in Fig. 3d and e, ⅳ–ⅵ, and h), while most PGRN-only positive cells in the boundary region were CD68 negative (open arrowheads in Fig. 3e, ⅳ–ⅵ). These data suggest that PSAP and PGRN coexpression mainly occurs in activated microglia/macrophages. To identify the cell type of cells positive only for PGRN, we performed co-immunostaining for PGRN, CD68, and various cell markers, such as the neuron marker TUJ1, the astrocyte marker S100B, and the oligodendrocyte marker PLP1. The PGRN-only cells co-localized with TUJ1, but not with S100B and PLP1, indicating neuron-like properties (Supplemental Fig. S5b–d). These findings indicate that as polydipsia progresses, active microglia expressing PSAP and PGRN infiltrate the area around the SFO.

### 2.4 Lysosomal PSAP and PGRN levels

**Fig. 4:**
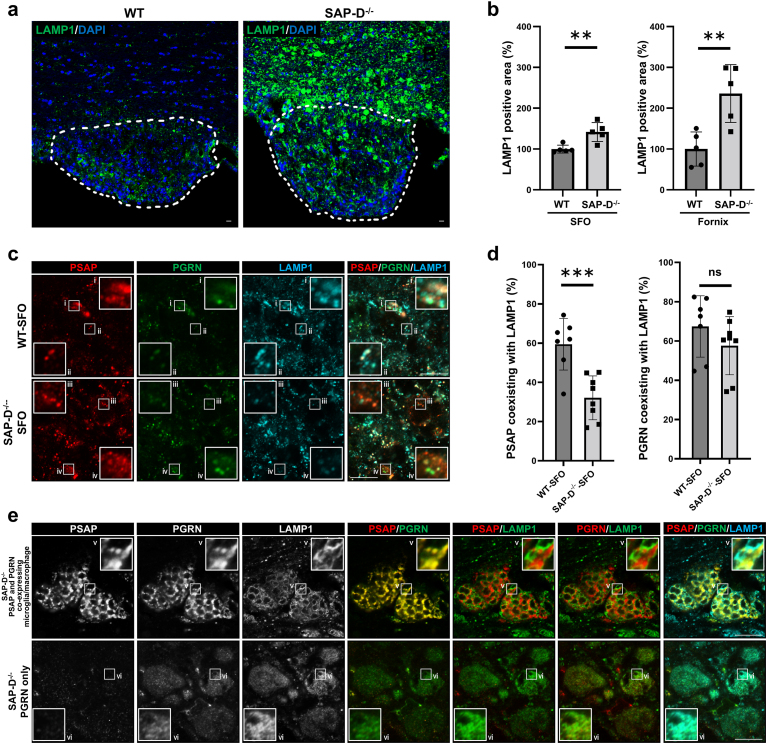
Lysosomal localization of PSAP and PGRN expression around the SFO **a)** Immunofluorescence staining of LAMP1 (green) around the SFO in 10-month-old female WT and SAP-D^−/−^ mice. Nuclei are labeled by DAPI (blue) staining. **b)** Quantification of LAMP1-stained areas in (**a**) relative to WT (%). The results of Student's t-tests for each panel are as follows: left panel, *p* = 0.0059, Cohen's *d* = 2.35 (95 % CI: 15.89, 67.66); and right panel: *p* = 0.0061, Cohen's *d* = 2.33 (95 % CI: 51.06, 220.89). Data are shown as the mean ± SD (n = 5). **c)** Triple immunofluorescent staining of PSAP (red), PGRN (green), and LAMP1 (cyan) around the SFO of 10-month-old female SAP-D^−/−^ mice. Enlarged images indicated by the white squares (ⅰ-iv) were shown in c. **d)** Localization rate of PGRN or PSAP to LAMP1 in the SFO. The results of Student's t-tests for each panel are as follows: left panel, *p* = 0.0008, Cohen's *d* = 2.24 (95 % CI: -40.90, -13.73); and right panel, *p* = 0.2341, Cohen's *d* = 0.64 (95 % CI: 26.80, 7.17). Data are shown as the mean ± SD (n = 6 for WT-SFO and n = 8 for SAP-D^−/−^-SFO). **e)** Same experiment as in (c) on microglia/macrophage co-expressing PSAP and PGRN, or PGRN only, in the boundary and fornix regions of SAP-D^−/−^ mice. Single image for each antibody was shown in white, while double or triple merged images were presented with red (PSAP or PGRN), green (LAMP1 or PGRN), or cyan (LAMP1) as indicated.  and  indicate the individual values in each group (b and d). ns: no significant difference. ∗∗∗*p* < 0.001. ∗∗*p* < 0.01. All scale bars, 10 μm.

Since PSAP and PGRN are lysosomal proteins, we performed co-immunostaining with LAMP1, a lysosomal marker. Interestingly, a dramatic increase of LAMP1 staining was observed in the SFO and the fornix of SAP-D^−/−^ mice compared to WT (Fig. 4a and b, and Supplemental Fig. S10), indicating that lysosomal biogenesis is promoted. Co-immunostaining of LAMP1 with PSAP and/or PGRN showed a significantly lower rate of PSAP and LAMP1 co-labeling from 59 % (WT) to 32 % (SAP-D^−/−^), whereas those of PGRN with LAMP1 were unchanged (67 % in WT and 57 % in SAP-D^−/−^; additional details of the statistical analysis are shown in the legend of Fig. 4c and d). In the boundary region and the fornix of SAP-D^−/−^ mice, LAMP1 staining was strong in microglia/macrophages expressing PSAP and PGRN, as well as in PGRN-only positive cells (Fig. 4e). In those regions, most PGRN signals co-localized with LAMP1 in cells expressing PGRN alone (Fig. 4e–vi). In cells expressing both PSAP and PGRN (square labeled “v” in Fig. 4e), many spots stained with both PSAP and PGRN—but not—LAMP1 were observed, suggesting that PGRN and LAMP1 co-localization clearly decreased in cells expressing both PSAP and PGRN (Fig. 4e–v). These results indicate that in SAP-D^−/−^ mice PSAP might partially inhibit not only its own migration to the lysosome but also that of PGRN. Thus, it was speculated that microglia strongly expressing PSAP and PGRN may have impaired lysosomal transport of these proteins.

### 2.5 Effects of PSAP expression on the water drinking behavior marker, c-Fos-positive cells

**Fig. 5:**
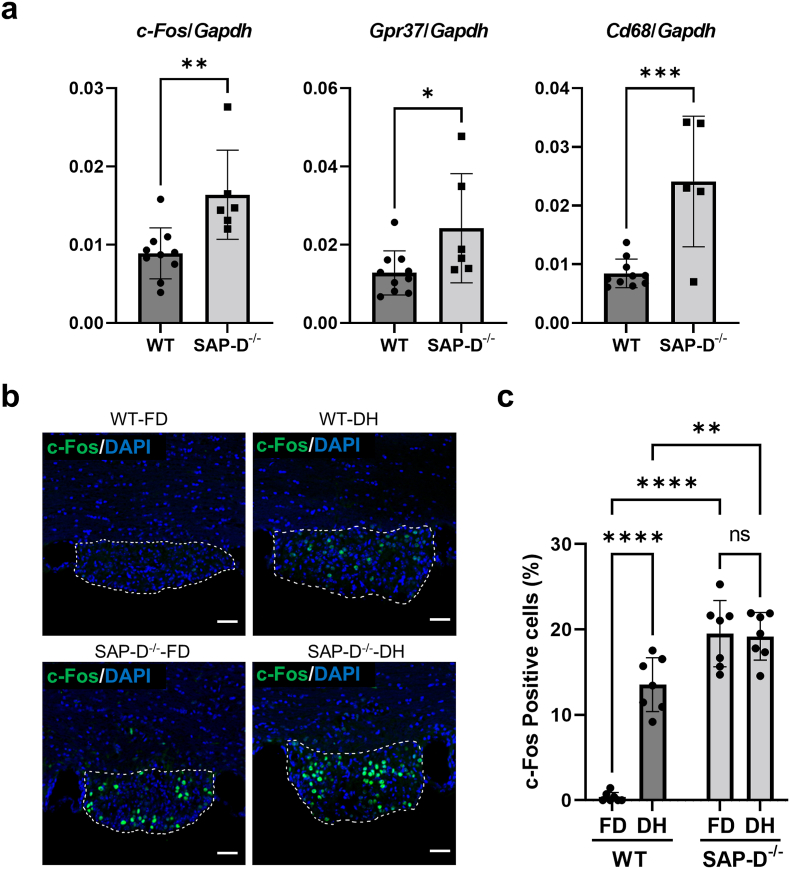
c-Fos expression was induced in the SFO of SAP-D^−/−^ mice without dehydration. a) Expression levels of *c-Fos*, *Gpr37*, and *Cd68* in the SFO and fornix were quantified via RT-qPCR. For RNA extraction, tissue samples were microdissected from the brains of 6-month-old female WT mice (n = 10) and SAP-D^−/−^ mice (n = 6) under a stereomicroscope. After cDNA synthesis, RT-qPCR was performed, and the data were normalized to *Gapdh* expression. A significant increase in *Cd68* expression was observed in SAP-D^−/−^ mice, consistent with immunostaining results, confirming accurate sampling of the SFO and surrounding fornix. The results of Student's t-tests for each panel are as follows: left panel, *p* = 0.0046, Cohen's *d* = 1.74 (95 % CI: 0.0027, 0.0122); center panel, *p* = 0.0352, Cohen's *d* = 1.20 (95 % CI: 0.00091, 0.02191); and right panel, *p* = 0.0007, Cohen's *d* = 2.41 (95 % CI: 0.0079, 0.0233). **b)** Immunofluorescent staining of c-Fos (green) in the SFO of 10-month-old female WT and SAP-D^−/−^ mice. WT and SAP-D^−/−^ mice had free access to drinking water (indicated as FD) or 24 h water deprivation (indicated as DH). The white dotted lines enclose the SFO. Nuclei are labeled by DAPI (blue) staining. Scale bars, 50 μm. **c)** Percentage of c-Fos positive cells among all DAPI stained cells in the SFO (%). Data are shown as the mean ± SD (n = 7). , , , and  indicate the individual values in each group. Two-way ANOVA showed significant main effects of water deprivation, F(1,24) = 35.13, *p* < 0.0001, ηp^2^ = 0.13, 95 % CI (−8.67, −4.19), and genotype, F(1,24) = 130.40, *p* < 0.0001, ηp^2^ = 0.36, 95 % CI (−14.64, −10.16), as well as a significant water deprivation × genotype interaction, F(1,24) = 38.60, *p* < 0.0001, ηp^2^ = 0.14, 95 % CI (−17.98, −9.01). Post-hoc Tukey's tests showed significant differences between WT-FD versus WT-DH (*p* < 0.0001, Cohen's *d* = 5.83 [95 % CI: −17.42, −8.94]), WT-FD versus SAP-D^−/−^-FD (*p* < 0.0001, Cohen's *d* = 6.91 [95 % CI: −23.38, −14.91]), and WT-DH versus SAP-D^−/−^-DH (*p* = 0.006, Cohen's *d* = 1.90 [95 % CI: −9.89, −1.41]), but no significant difference between SAP-D^−/−^-FD versus SAP-D^−/−^-DH (*p* = 0.997, Cohen's *d* = 0.09 [95 % CI: −3.92, 4.54]). ns: no significant difference. ∗∗∗*p* < 0.001, ∗∗*p* < 0.01, ∗*p* < 0.05.

c-Fos is known as a marker of exciting neuron. Because c-Fos expression in nuclei in the SFO is strongly associated with drinking water behavior [48,49], the relationship between c-Fos expression and PSAP and/or PGRN in SAP-D^−/−^ mice was investigated. RT-qPCR analysis (Fig. 5a) revealed significantly elevated c-Fos expression along with significantly increased GPR37 expression, with GPR37 being a receptor known to bind to extracellular PSAP and activate ERK, an upstream regulator of c-Fos gene expression [[50], [51], [52]].

Immunofluorescence revealed that in WT mice, the rate of c-Fos-positive cells in the SFO was 0.4 % under the condition of free drinking (FD); however, the number of c-Fos-positive cells in the SFO remarkably increased to 13.5 % after 24 h of water deprivation, (dehydration condition, DH), which should induce thirst, suggesting that DH induced c-Fos expression in the SFO as well as thirst (Fig. 5b and c). In SAP-D^−/−^ mice, the rate of c-Fos-positive cells was approximately 20 % under FD and under DH, which was higher than that in dehydrated WT mice (Fig. 5b and c). Meanwhile, in 1-month-old female SAP-D^−/−^ mice before the onset of polydipsia, little c-Fos expression was observed at the same level as WT under FD (Supplemental Figs. S4c and S11). Age-progression analysis showed a significant rise in c-Fos expression from 3 months of age, when polydipsia and polyuria begin, to 10 months of age (Supplemental Fig. S11). In addition, 90 % of c-Fos-positive cells in the SFO of 10-month-old SAP-D^−/−^ mice were positive for TUJ1, indicating their neuronal identity (Supplemental Fig. S13). These results demonstrate that the rate of c-Fos expressing cells is in line with the water-drinking behavior in WT and SAP-D^−/−^ mice. Taken together, they suggest that infiltration of microglia strongly expressing PSAP/PGRN induces c-Fos expression, potentially promoting polydipsia onset and progression.

## 3 Discussion

In this study, we identified activated microglia/macrophages with high PSAP and PGRN expression infiltrating the SFO in SAP-D^−/−^ mice as polydipsia progressed. These microglia exhibited impaired PSAP and PGRN transport to lysosomes, leading to intracellular accumulation. Concurrently, c-Fos-positive cell counts, a marker of drinking behavior, significantly increased. These findings suggest that infiltration of microglia strongly expressing PSAP and PGRN around the SFO triggers polydipsia via c-Fos induction. Notably, this is the first report linking PSAP to polydipsia.

### 3.1 Accumulation of PSAP and PGRN around the SFO in SAP-D^−/−^ mice

In SAP-D^−/−^, PSAP was expressed throughout the brain, including the cerebral cortex and the SFO, whereas PGRN was strongly expressed around the SFO (Fig. 1a–h). Regarding the role of these molecules in the brain, it has been reported that their precise intracellular and extracellular transport is important for normal cognitive function and that several mutations in the gene encoding PGRN or PSAP cause various neurodegenerative diseases such as dementia and Parkinson's disease [16,17,53,54]. Although both PSAP and PGRN protein levels are decreased in the brains of patients with schizophrenia [55], in some reports both are increased in Alzheimer's disease [14]. Increased PGRN expression levels have also been observed in some neurodegenerative diseases, regardless of PSAP reduction or overexpression [14,56]. Thus, it is possible that the accumulation of both in the CVO, especially in and around the SFO, might be closely related to the abnormal drinking behavior in SAP-D^−/−^ mice.

At the cellular level, we observed strong coexpression of these two proteins in microglia/macrophages (Fig. 3). Secreted PSAP from microglia exerts physiological effects on other cells [51,57,58]. The infiltration of PSAP/PGRN-expressing microglia alongside polydipsia onset suggests that these proteins may play a prominent role in the disease's progression.

### 3.2 PSAP and PGRN levels in the lysosome

PSAP binds to PGRN and their intracellular and extracellular transport requires each other [[23], [24], [25], [26]]. Those proteins have been also reported to cooperate each other to maintain lysosome function normally [14,16,58,59].

Lefrancois et al. reported that the SAP-D domain is required for the correct lysosomal trafficking of PSAP [60]. Oji et al. reported that mutations in the SAP-D domain of PSAP may result in failed PSAP trafficking to the lysosome and accumulation in the ER, which may contribute to the development of Parkinson's disease [61]. In this study, a certain number of PSAP in SAP-D^−/−^ mice did not colocalize with LAMP1 (Fig. 4c and d), suggesting an impairment in PSAP transport to the lysosome. Regarding PGRN, its LAMP1 localization rate was similar in the SAP-D^−/−^ and WT SFO (Fig. 4c and d). In the boundary and the fornix of SAP-D^−/−^mice, despite PGRN predominately localizing to LAMP1 in cells strongly expressing PGRN alone (Fig. 4e–vi), the co-localization rate of PGRN co-expressed with PSAP to lysosomes in microglia/macrophages was notably reduced (Fig. 4e–v). This suggests that mutant PSAP impairs its own and PGRN's lysosomal translocation. Additionally, the reason might be that, as shown in Supplemental Fig. S14, the amount of sortilin, which transports PSAP/PGRN to lysosomes [25,62,63], did not increase despite PSAP/PGRN overexpression, remaining at WT levels.

PSAP is involved in lysosome maintenance [11,64]. We showed that the amount of lysosome was increased in SAP-D^−/−^ (Fig. 4a and b), speculating that biosynthesis of lysosome is upregulated to compensate for lysosomal dysfunction. Normally, PGRN and PSAP are degraded to SAPs and GRNs after reaching the lysosomes, which are released and should not be stored in lysosomes. Given these findings, the following assumptions can be made: in SAP-D^−/−^ mice, it is difficult for PSAP to reach the lysosome where it is processed, resulting in accumulation in the cell. When coexpressed with PSAP, PGRN behaves in a similar way as PSAP; however, when expressed on its own, it is transported to lysosomes. Further research, including *in vitro* experiments, is needed to confirm this hypothesis. We also observed an increased number of lysosomes in SAP-D^−/−^ mice (Fig. 4a and b). Within lysosomes, the degradation of glycosphingolipids (GSLs) involves SAPs cleaved from PSAP [11,64]. In SAP-D^−/−^ mice, GSL degradation function is thought to be impaired due to the absence of SAP-D and the difficulty of PSAP reaching the lysosomes. Consequently, lysosomal biogenesis may be enhanced as a compensatory mechanism.

### 3.3 c-Fos expression and polydipsia

As shown in Fig. 5, a large number of c-Fos-positive cells were observed under FD and DH in SAP-D^−/−^ mice, suggesting a correlation with the extent of drinking behavior. We also showed that 90 % of c-Fos positive cells were positive for the neuronal marker TUJ1, confirming their neuronal identity (Supplemental Fig. S13). Thus, c-Fos-positivity might be key for polydipsia. The correlation between increased c-Fos-positive cells and polydipsia progression suggests a link with the infiltration of microglia strongly expressing PSAP/PGRN.

As shown in Supplemental Fig. S14, sortilin expression remained unchanged despite elevated PSAP/PGRN levels, indicating that substantial amounts bypass lysosomes and are potentially excreted extracellularly [22,25]. We hypothesize that secreted PSAP/PGRN stimulates neurons, inducing c-Fos expression and promoting polydipsia. Increased GPR37 expression supports this hypothesis, as secreted PSAP binds to the GPCR receptor GPR37, activates intracellular signaling, and promotes c-Fos expression via ERK phosphorylation [[50], [51], [52]]. *GPR37* gene expression around the SFO was significantly higher in SAP-D^−/−^ mice than in WT mice (Fig. 5a), suggesting that PSAP secretion from microglia activates ERK via GPR37, inducing c-Fos expression and excessive water intake, ultimately driving polydipsia onset and progression.

In Evans blue injection experiments (Supplemental Fig. S15), dye spread beyond the fornix in SAP-D^−/−^ mice, whereas in WT mice, it remained localized to the SFO [65]. This suggests the occurrence of blood–brain barrier disruption, making the SFO more susceptible to stimulation of c-Fos expression via PSAP and other humoral stimuli, leading to sustained drinking behavior.

Further analysis of c-Fos-positive cells (Supplemental Fig. S12a and b) showed significantly higher PSAP expression in c-Fos-positive vs. c-Fos-negative cells in SAP-D^−/−^ mice under both FD and DH conditions, a pattern absent in WT. However, PGRN expression did not correlate with c-Fos levels in WT or SAP-D^−/−^ animals. Additionally, c-Fos was absent in CD68-expressing cells (Supplemental Fig. S12c). DH did not affect PSAP/PGRN protein expression (Supplemental Fig. S16). These findings suggest that PSAP accumulation in neurons influences c-Fos expression and, consequently, water-drinking behavior. Further isolation of c-Fos-positive cells and a comprehensive expression analysis could identify relevant factors and elucidate the underlying mechanism of c-Fos expression under these conditions.

In summary, we found that in SAP-D-deficient mice, the infiltration of microglia/macrophage strongly enriched with PSAP and PGRN increased in parallel with the progression of polydipsia and with the expression of c-Fos, which is deeply associated with drinking behavior. These findings suggest that PSAP and PGRN strongly accumulated in microglia may contribute to the development of polydipsia. These findings highlight a potential mechanism underlying polydipsia in SAP-D^−/−^ mice.

## 4 Methods

### 4.1 Animals

All animal experiments were conducted with the approval of the Teikyo University Animal Care and Use Committee (2206362A1b & 22-012) and in compliance with the ARRIVE guidelines. SAP-D deficient (SAP-D^−/−^) mice carrying a missense mutation (p.C509S) in the SAP-D domain of murin PSAP were previously generated using Cre/loxP gene targeting strategy [8]. SAP-D (amino acids 438–519) was generated by processing from the 557-amino acid PSAP protein. The C509S mutation caused SAP-D dysfunction by deletion of the intramolecular disulfide bond with the cysteine residue at 445 (Supplemental Fig. S17). Previous reports using a specific anti-SAP-D antibody and our immunoblotting data using anti-human SAP-D antibody (431 003, Synaptic Systems GmbH, G31 003, S Germany) illustrated that SAP-D production was absent in the brains of SAP-D^−/−^ mice (Supplemental Fig. S2a), suggesting that the C509S mutation is important in the processing of PSAP to SAP-D [9,10]. WT and SAP-D^−/−^ mice were bred and handled according to Teikyo University guidelines for the use and care of experimental animals, as accredited by the Japanese Ministry of Education, Culture, Sports, Science, and Technology. Experiments were carried out in 3, 6, and 10-month-old WT, and SAP-D^−/−^ mice. Mice had free access to tap water or were water-deprived for 24 h to cause dehydration. One-month-old mice were used to examine PSAP and PGRN expression before the onset of polydipsia.

### 4.2 Protein isolation

A cerebral region containing the SFO (0.7–0.8 mm posterior to bregma) was sliced to a thickness of 1 mm using Brain Slicer Matrix (EMJapan co., Ltd., Japan; Fig. 1c). Sliced brains were immediately frozen in liquid N_2_ and stored at −80 °C. Protein extraction from the brain samples was performed as previously described [31].

### 4.3 Western blotting

Protein extracts were subjected to western blotting as described before [31] and expressed relative to GAPDH. Polyacrylamide gel (5 %–20 %) and PVDF membranes (ATTO co., Japan) were used for SDS-PAGE and blotting. Membranes were blocked with 5 % nonfat milk in TBS-T (1 % Tween 20) overnight at 4 °C and incubated in the appropriate primary antibody in 3 % BSA in TBS-T for 2 h at room temperature shaking with PSAP (1:1,000, 10801-1-AP, Proteintech, USA), PGRN (1:1,000, AF 2557, R&D Systems, USA), and GAPDH (1:5000 60004-1-Ig, proteintech, USA). After washing, membranes were incubated in the appropriate HRP-conjugated secondary antibody (1:5,000, sc-2357 or 1:10 000 7076 CST, USA) for 1 h at room temperature, and signals were detected with Ez-West LumiOne or EZWestLumi plusand (ATTO co., Japan). Visualization and quantification of each signal were performed using ChemiDoc XRS Plus and Image Lab ver. 5.0 (Bio-Rad Laboratories, Inc., USA). Full scale western blots are shown in Supplemental Figs. S18–S21.

### 4.4 Immunofluorescent staining

All 10-month-old female mice, both WT and SAP-D^−/−^, were anesthetized with isoflurane and reflux-fixed in 4 % paraformaldehyde in 50 mM phosphate-buffer, pH 7.5, (PFA), following refluxing with 50 mM phosphate-buffered saline, pH 7.5 (PBS). Subsequently, their brains were extracted and immersion-fixed in 4 % PFA for 48 h, followed by substitution with 30 % sucrose in PBS and embedding in Tissue Tek OCT (Sakura Finetek USA, CA, USA) for cryopreservation. Next, 10 μm frozen coronal sections containing the SFO were obtained using a Leica CM3050 S cryostat (Leica Microsystems GmbH, Wetzlar, Germany). The sections were permeabilized and blocked with 10 % fetal bovine serum in PBS-T (0.4 % Triton-X) for 1 h at room temperature, followed by incubation with primary antibodies at the appropriate dilution overnight at 4 °C. The primary antibodies used were a rabbit antibody against PSAP (dilution 1:100, 10801-1-AP, Proteintech Group Inc., IL, USA), a sheep antibody against PGRN (dilution 1:100, AF 2557, R&D Systems Inc., MN, USA), a guinea pig antibody against c-Fos (dilution 1:500, 226308, Synaptic Systems GmbH, Göttingen, Germany), a rat antibody against CD68-FITC conjugated (dilution 1:500, MCA1957FA, BIO-RAD Laboratories Inc., CA, USA), and a rat antibody against LAMP1 (dilution 1:100, ab25245, Abcam, Cambridge, UK). After washing in PBS, incubation with secondary antibodies was performed using Alexa 488-, Cy3-or Alexa 647-conjugated AffiniPure donkey anti-guinea pig, -rabbit, -sheep, or rat IgG (dilution 1:1,000, Jackson ImmunoResearch Laboratories, Inc. PA, USA), respectively. The sections were washed in PBS and enclosed in Vectashield Mounting Medium with 4′,6-diamidino-2-phenylindole (DAPI) (Vector, Burlingame, CA, USA). Immunofluorescence was observed under a fluorescence microscopy with a BZ-X800 (KEYENCE, Osaka, Japan) and confocal laser microscopy with a LSM880 (Leica Microsystems GmbH). All fluorescent images were acquired as black-and-white photographs and presented in false-colors. All DAPI staining is shown in blue. The signal areas of immunofluorescence staining (Fig. 3b, d, 3e, 3f, S7b, S7d, and S9b) were quantified using BZ-X Analyzer (KEYENCE). The co-labeling rate for each immunofluorescence signal (Fig. 4b and d) and the intensity of immunofluorescence staining for individual cells (Supplemental Fig. S12a and b) were quantified using ZEN Microscopy Software (Leica Microsystems GmbH).

### 4.5 RT-qPCR

For RNA extraction, the SFO and surrounding fornix were microdissected from the brains of 6-month-old female mice under a stereomicroscope. RNA was purified using the RNeasy Micro Kit (QIAGEN K.K., Tokyo, Japan) following the manufacturer's protocol. Given the limited RNA yield, cDNA synthesis was performed using the PrimeScript™ RT Reagent Kit (Takara Bio, Inc.). Real-time quantitative PCR (RT-qPCR) was conducted using the Thermal Cycler Dice® Real Time System III (Takara Bio Inc., Shiga, Japan) and TB Green® Premix Ex Taq TMII (Takara Bio, Inc.). Gene expression levels were normalized to *Gapdh* and presented as relative expression values. The primer sequences used for each gene are listed in Supplemental Table S1.

### 4.6 Statistical analysis

One-factor analysis of variance (one-way ANOVA) with Tukey's test (Fig. 1h), two-way analysis of variance (two-way ANOVA) with Tukey's test (Fig. 1e, f, 5c and Supplemental Fig. S8c), and Student's t-test (Fig. 4b, d, 5a, S1, S3, S4b, S11b, S12b, S14b, S14d and S16b) were used to analyze the data. For one-way and two-way ANOVA, partial eta-squared (ηp^2^) and the 95 % CI were calculated as measures of effect size. For Student's t-tests, Cohen's *d* and the 95 % CI were calculated as measures of effect size. The sample number in each experiment is indicated in each figure legend. All graphs were created using GraphPad Prism 9 (GraphPad Software, Inc. CA, USA).

### Limitations of the study

This study demonstrated that PSAP accumulated in microglia/macrophages might cause polydipsia in SAP-D-deficient mice. However, direct evidence supporting this hypothesis has not yet been established. This represents the limitation of the present study.

## Author contributions

HH, TS, TO, and MTA conceived the research idea. HH, TS, and MTA designed the methodology. HH, TS, NO, MA, TO, and MTA carried out the experiment and analyzed the data. MI, JM, SU, and HO contributed to the interpretation of the result. All authors discussed the results and commented on the manuscript. HH, TS, TO, and MTA wrote the manuscript with input from all authors.

## Ethics declaration

All animal studies were approved by the Teikyo University Animal Care and Use Committee (2206362A1b & 22-012) and were conducted in accordance with relevant institutional guidelines and regulations. The study also adhered to the ARRIVE guidelines.

## Declaration of competing interest

All authors declare that they have no competing interest for the current work.

## Data Availability

Data will be made available on request.
