# Supplementary material for: Accumulation of prosaposin and progranulin around the subfornical organ induces polydipsia in SAP-D-deficient mice
Source: Biochem Biophys Rep. 2025 Dec 2;45:102388. doi: 10.1016/j.bbrep.2025.102388 (PMC12720004; doi:10.1016/j.bbrep.2025.102388)
Supplement: Multimedia component 1 [file mmc1.pdf]

Supplemental Information

Accumulation of prosaposin and progranulin around the subfornical organ induces polydipsia in SAP-D-deficient mice

Harumi Hisaki[1], Takao Susa[1]**, Noriyuki Okudaira[1], Miho Akimoto[1], Masayoshi Iizuka[1,2], Junko Matsuda[3], Shunya Uchida[4], Hiroko Okinaga[5], Tomoki Okazaki[1], and Mimi Tamamori-Adachi[1]*

[1] Department of Biochemistry, Teikyo University School of Medicine, Itabashi, Tokyo, Japan

[2] Center for Medical Education, Teikyo University, Itabashi, Tokyo, Japan

[3] Department of Pathophysiology and Metabolism, Kawasaki Medical School, Okayama, Japan

[4] Teikyo Heisei University, Toshima, Tokyo, Japan

[5] Department of Internal medicine, Teikyo University School of Medicine, Itabashi, Tokyo, Japan

***Correspondence**: madachi@med.teikyo-u.ac.jp

** **Correspondence**: tsusa@med.teikyo-u.ac.jp

Supplemental Table S1. Primer sets used for RT-qPCR

Gene	Forward Primer	Reverse Primer
Psap	CCAAGACCCGAAGACATGCT	AGGAAGGGATTTCGCTGTGG
Pgrn	GCCTGGAGAAGATACCTGCC	CTCACAGCACACAGCATGG
Cd68	TCTCTAAGGCTACAGGCTGCT	CAATGATGAGAGGCAGCAAG
Gpr37	TGACTAAGAAGTGGCTTTTGGA	GCACAGTGCACATAAGGTGA
c-Fos	GGAGGACCTTACCTGTTCGTGA	GAACAACACACTCCATGCGG
Gapdh	GGTTGTCTCCTGCGACTTCA	TAGGGCCTCTCTTGCTCAGT

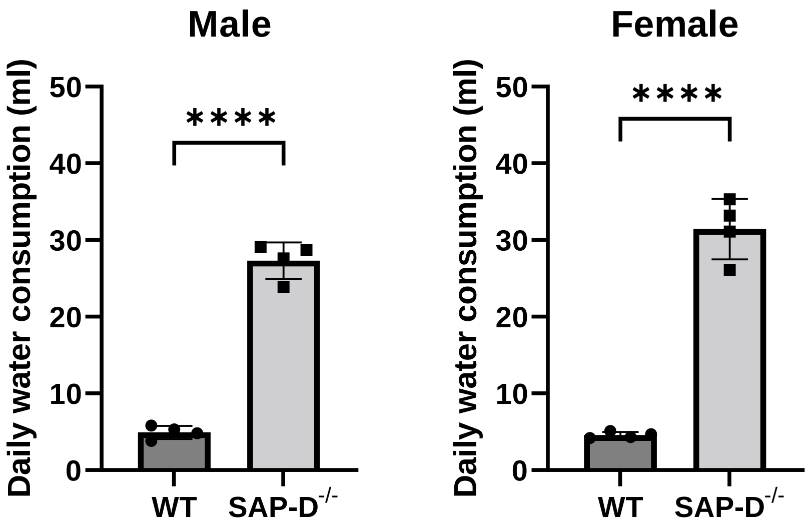

Figure S1. Water intake increases in 10-month-old male and female SAP-D$^{-/-}$ mice

Daily water intake data are shown. The increase in water intake in SAP-D$^{-/-}$ mice was reproducible. Comparisons between two groups were performed using Student's t-test. The results for each panel are as follows: males, $p < 0.0001$, Cohen's $d = 12.57$ (95% CI: 19.31, 25.48); and females, $p < 0.0001$, Cohen's $d = 9.57$ (95% CI: 22.00, 31.69). Bars indicate the mean ± SD of 4 mice per group. Circles and squares represent individual mice.

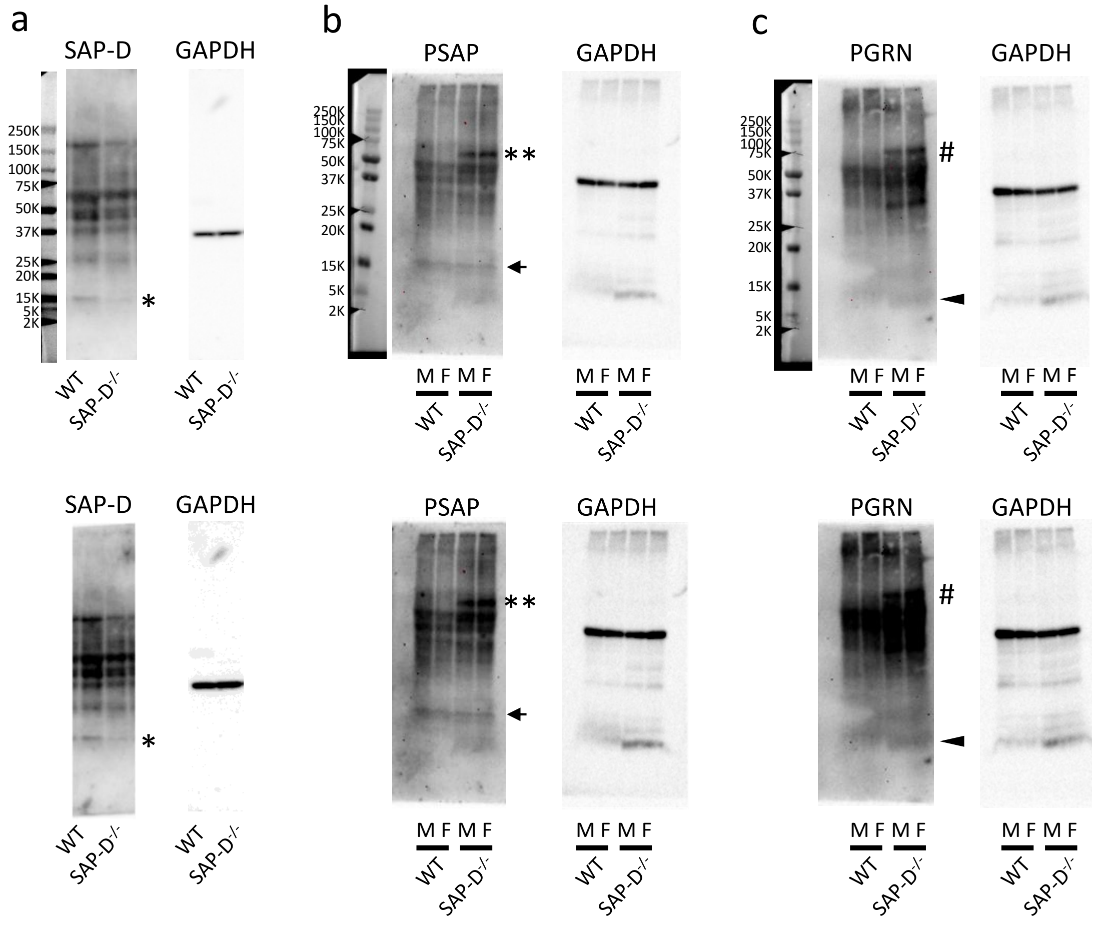

Figure S2. Saposins and granulins by immunoblotting in WT and SAP-D$^{-/-}$ mice

The cerebral region containing the SFO (0.7–0.8 mm posterior to bregma) was used for protein extraction and immunoblotting. SAP-D was electrophoresed on a 5%–20% glycine gradient gel (a), and both PSAP (b) and PGRN (c) were electrophoresed on a 15% glycine gel. a) Samples containing the SFO were prepared from 3-month-old female mice. Anti-SAP-D antibody (Cat. No. 431 003, Synaptic Systems, Göttingen, Germany) was used to confirm the absence of SAP-D in SAP-D$^{-/-}$ mice. The band corresponding to SAP-D was denoted by an asterisk (*). b–c) Protein samples were prepared from 11-month-old male and female mice. b) The PSAP and saposins (SAPs) bands as detected by immunoblotting using anti-PSAP antibody are indicated by asterisks (**) and an arrow (14 kDa), respectively. c) The PGRN and granulins (GRNs) bands as detected by immunoblotting with anti-PGRN antibody are indicated by the pound sign (#) and an arrowhead (8 kDa), respectively. M, male; F, female, Immunoblotting with anti-GAPDH antibody was performed as a protein loading control. Long-exposure photographs are presented below the blots for each panel.

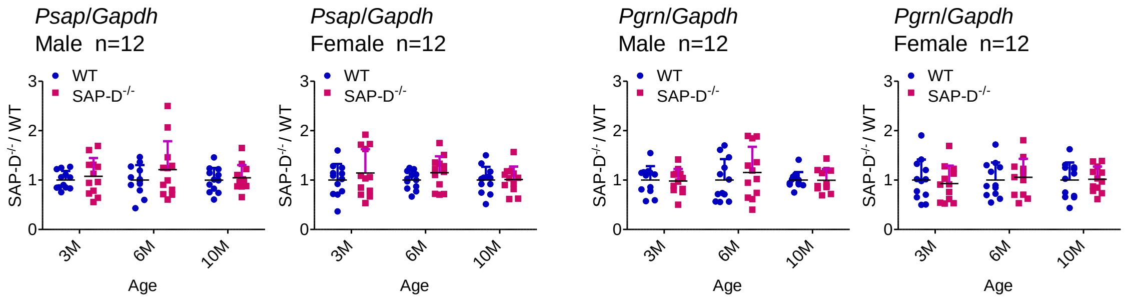

Figure S3. RT-qPCR analysis of *Psap* and *Pgrn* expression in the SFO at the indicated age groups

Cerebral region from 3-, 6-, and 10-month-old male and female mice containing the SFO (0.7–0.8 mm posterior to bregma, as shown in Figure 1c) was used for RNA extraction. RNA extraction was performed by using RNAiso plus (Takara Bio, Inc., Shiga, Japan) according to the manufacture's protocols. cDNA synthesis was performed using the PrimeScript RT reagent kit (Takara Bio, Inc.). Quantitative PCR was performed using the Thermal Cycler Dice Real Time System III (Takara Bio Inc., Shiga, Japan) and TB Green Premix Ex Taq TMII (Takara Bio, Inc.). The primer sequences for each gene are listed in Table S1. Gene expression of *Psap* and *Pgrn* was normalized by *Gapdh* and presented as a ratio to that in 3-month-old wild-type (WT) mice. Statistical analysis between the two groups was performed using Student's t-tests. The results for each panel are as follows: PSAP-Male-3M, p = 0.5222, Cohen's d = 0.26 (95% CI: −0.16, 0.32); PSAP-Male-6M, p = 0.2371, Cohen's d = 0.45 (95% CI: −0.17, 0.59); PSAP-Male-10M, p = 0.6576, Cohen's d = 0.18 (95% CI: −0.16, 0.26); PSAP-Female-3M, p =0.4037, Cohen's d = 0.34 (95% CI: −0.20, 0.49); PSAP-Female-6M, p = 0.1920, Cohen's d = 0.54 (95% CI: −0.07, 0.37); PSAP-Female-10M, p = 0.9121, Cohen's d = 0.04 (95% CI: −0.21, 0.23); PGRN-Male-3M, p = 0.8609, Cohen's d = 0.07 (95% CI: −0.24, −0.20); PGRN-Male-6M, p = 0.4359, Cohen's d = 0.32 (95% CI: −0.24, 0.55); PGRN-Male-10M, p = 0.9635, Cohen's d = 0.01 (95% CI: −0.17, 0.16); PGRN-Female-3M, p = 0.6602, Cohen's d = 0.18 (95% CI: −0.40, 0.25); PGRN-Female-6M, p = 0.7085, Cohen's d = 0.15 (95% CI: −0.25, 0.36); PGRN-Female-10M, p = 0.8969, Cohen's d = 0.05 (95% CI: −0.24, 0.27). No significant differences were observed between WT and SAP-D$^{-/-}$ at each month of age. Bars indicate the mean ± SD of 12 mice per group, and circles and squares represent individual mice.

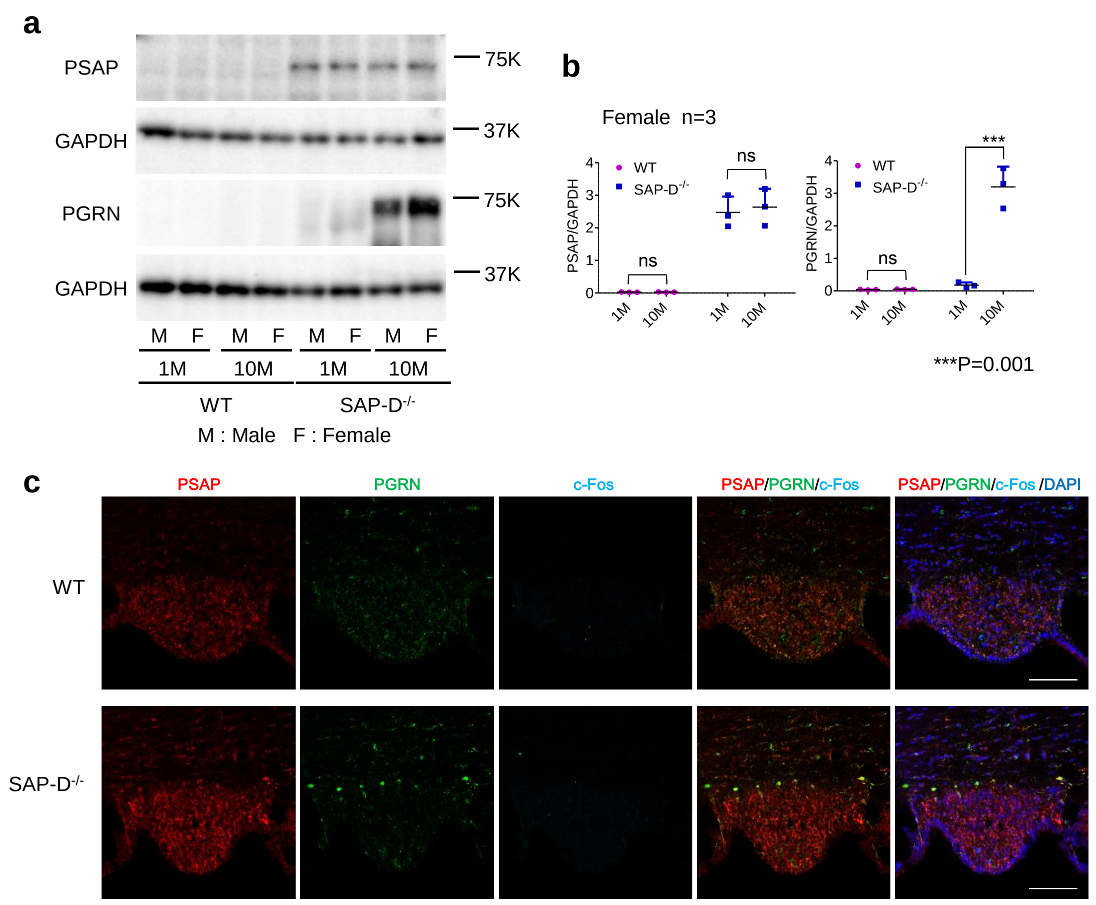

Figure S4. PSAP, PGRN, and c-Fos expression in the SFO of 1-month-old mice

a) Western blots of PSAP and PGRN at the indicated age. The cerebral region containing the SFO (0.7–0.8 mm posterior to bregma, as shown in Figure 1c) was used for protein extraction and immunoblotting. b) Quantification of the western blot data by densitometric analysis in female mice. Protein expression was normalized by GAPDH and presented as a ratio to that in 1-month-old. Bars indicate the mean ± SD of three mice per group, and circles and squares indicate individual mice. Statistical analysis was performed using Student's t-tests. The results for each panel are as follows: PSAP/GAPDH-WT, p = 0.8565, Cohen's d = 0.15 (95% CI: −0.013, 0.015); PSAP/GAPDH-SAP-D$^{-/-}$, p = 0.7243, Cohen's d = 0.30 (95% CI: −1.03, 1.35); PGRN/GAPDH-WT, p = 0.2061, Cohen's d = 1.23 (95% CI: −0.008, 0.027); PGRN/GAPDH-SAP-D$^{-/-}$, p = 0.0011, Cohen's d = 6.80 (95% CI: 2.01, 4.02). c) Triple immunofluorescence staining of PSAP (red), PGRN (green), and c-Fos (cyan) in the SFO of 1-month-old female mice. Nuclei were stained with DAPI (blue). Scale bar, 100 µm.

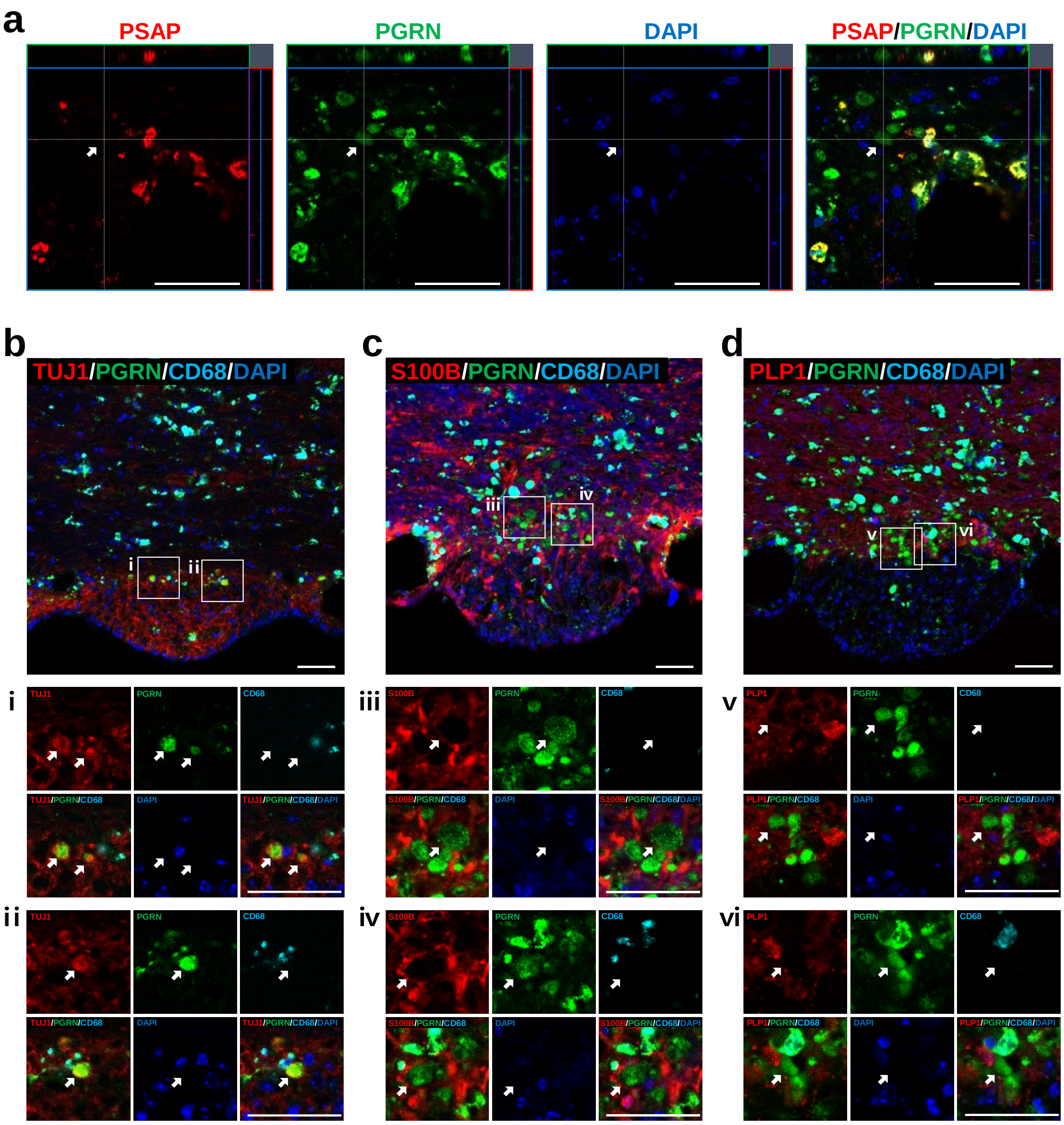

Figure S5. Advanced analysis of PGRN-only immunopositive cells in 10-month-old female SAP-D$^{-/-}$ mice

a) Three-dimensional analysis of PGRN-only immunopositivity by double staining for PSAP (red) and PGRN (green) around the SFO in 10-month-old female SAP-D$^{-/-}$ mice. Arrows indicate the location of PGRN-only cells. b–d) Analysis of PGRN-only cells exclusive of CD68 signals as detected by multiple immunostaining with various marker antibodies. b) Anti-beta tubulin 3 (TUJ1) antibody (ab14545, Abcam, Cambridge, UK; red) was used as a neuron-specific marker. Arrows indicate that the PGRN-only signals co-localized with TUJ1. c) Anti-S100B (#90393, Cell Signaling Technology, Inc., Danvers, MA, USA) antibody (red) was used as an astrocyte-specific marker. Arrows indicate that the PGRN-only signals do not co-localize with S100B. d) Anti-PLP1

antibody (#28702, Cell Signaling Technology, Inc.; red) was used as an oligodendrocyte-specific marker. Arrows indicate that the PGRN-only signals do not co-localize with PLP1. b–d) Enlarged white i–iv squares as indicated. The nuclei were stained with DAPI (blue) staining. The PGRN-only signals have no nuclei, as indicated by the absence of DAPI staining (blue). All scale bars indicate 50 µm.

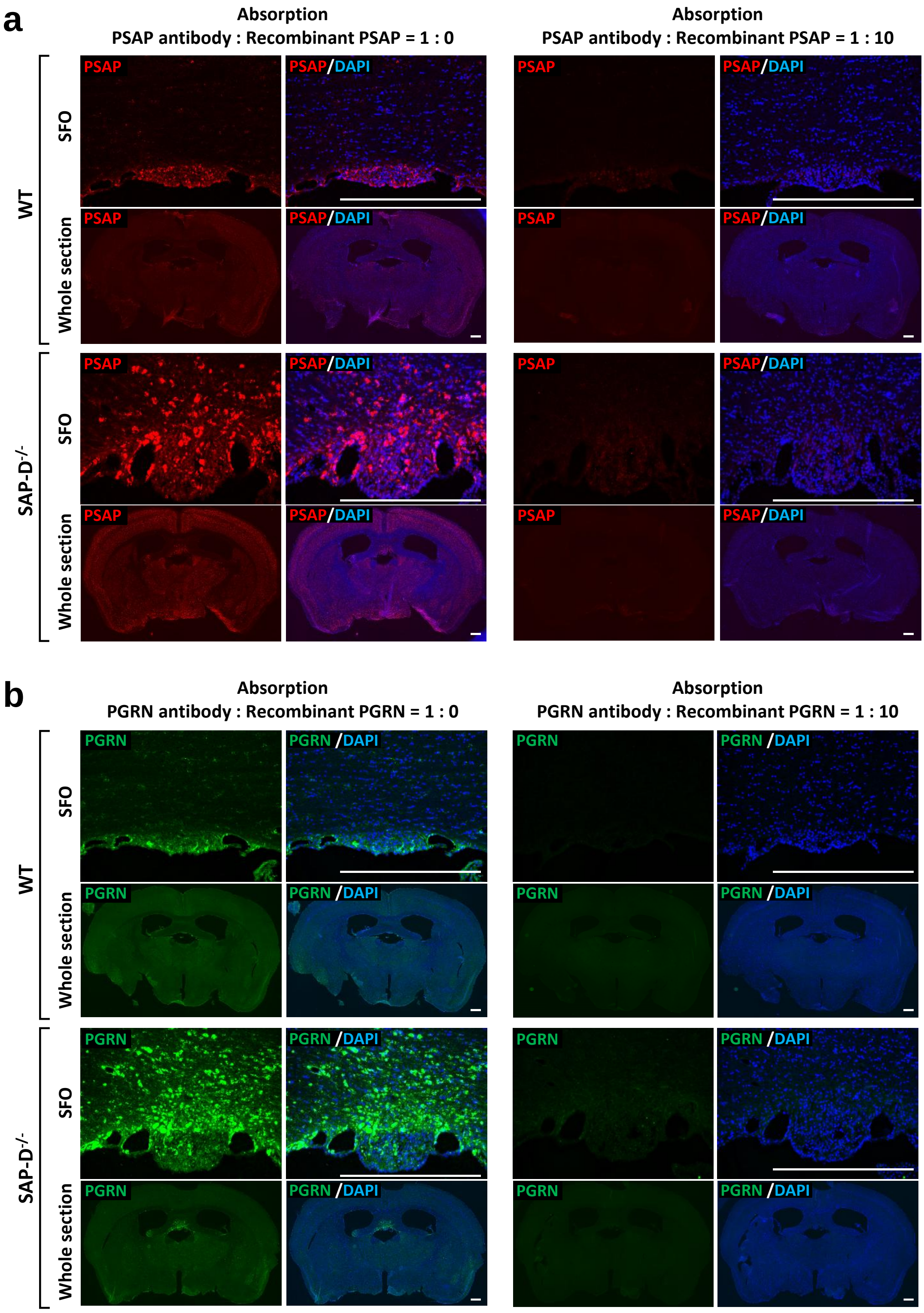

Figure S6. Immunofluorescence using absorbed PSAP or PGRN antibodies against specific antigens

The antigen specificities of PSAP (a) and PGRN (b) antibodies were verified by antigen absorption experiments. Recombinant PSAP (16224-H08H, Sino Biological, Inc.,

Beijing, China) and PGRN (2557-PG, R&D Systems, Inc., Minneapolis, MN, USA) were used. Immunofluorescence was performed via incubation with antibodies and antigens at a molar ratio of 1:10 for 6 h at room temperature. The nuclei were stained with DAPI (blue). All scale bars indicate 500 μm.

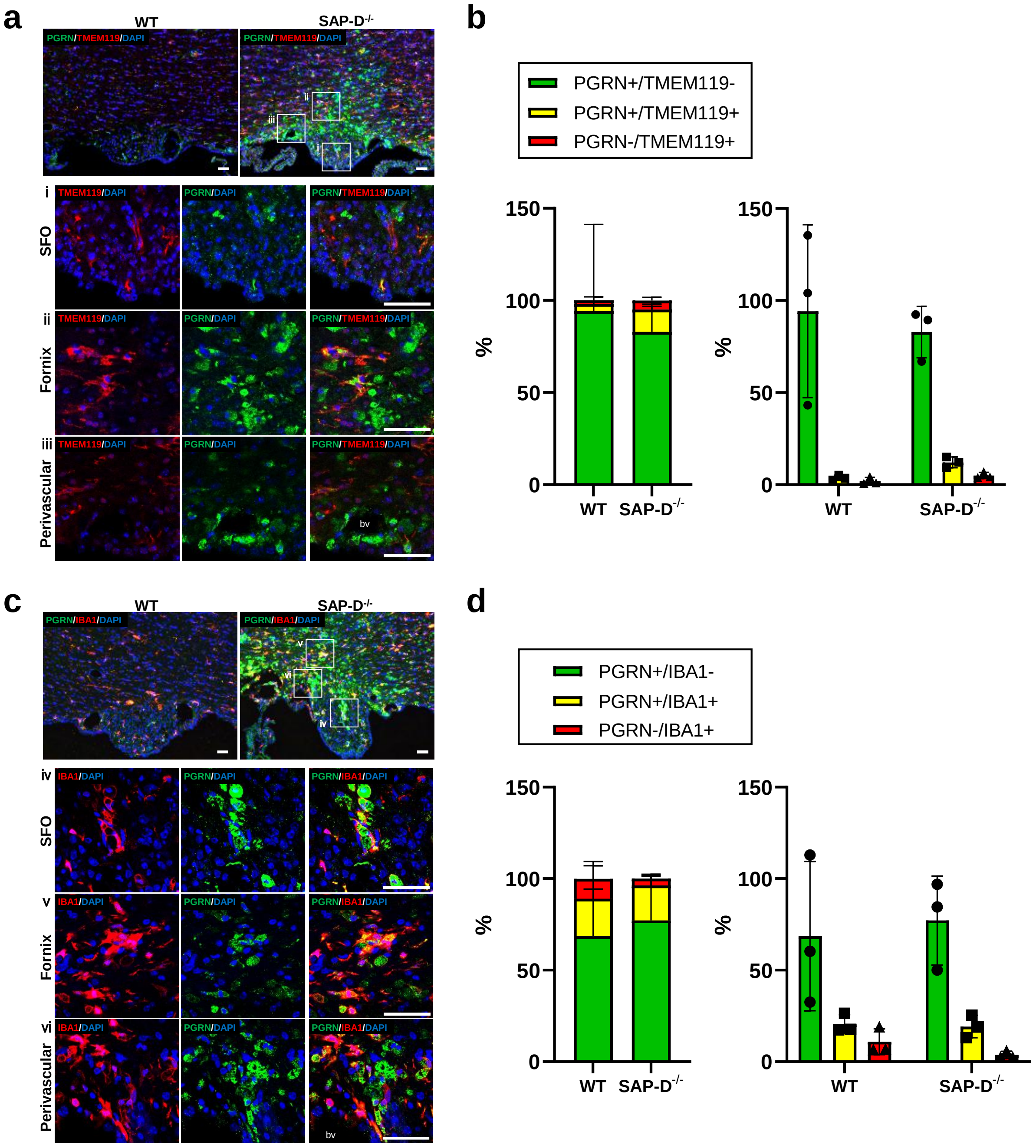

Figure S7. **Protein expression of TMEM119 and IBA-1 in the SFO of 10-month-old female mice**

a) Double immunofluorescence staining for PGRN (green) and TMEM119 (ab209064, Abcam, Cambridge, UK; red) around the SFO in 10-month-old female WT and SAP-D$^{-/-}$ mice. The white squares in SAP-D$^{-/-}$ mice were magnified in i–iii (i , SFO; ii , fornix; iii, perivascular). b) Quantification of PGRN- and/or TMEM119-positive areas in the SFO and surrounding areas of WT and SAP-D$^{-/-}$ mice (%). The left panel presents a stacked bar chart, whereas the right panel shows the individual data values in a bar chart format. Data are presented as the mean ± SD (n = 3). c) Double immunostaining for PGRN (green) and IBA-1 (GTX100042, Gene Tex, Inc., CA; red)

around the SFO in 10-month-old female WT and SAP-D$^{-/-}$ mice. The white squares in SAP-D$^{-/-}$ mice were magnified in iv–vi (iv, SFO; v, fornix; vi, perivascular). d) Quantification of PGRN- and/or IBA-1–positive areas in the SFO and surrounding areas of WT and SAP-D$^{-/-}$ mice (%). The left panel presents a stacked bar chart, whereas the right panel shows the individual data values in a bar chart format. Data are presented shown as the mean ± SD (n = 3). Nuclei are stained with DAPI (blue). bv: blood vessel. All scale bars indicate 50 µm.

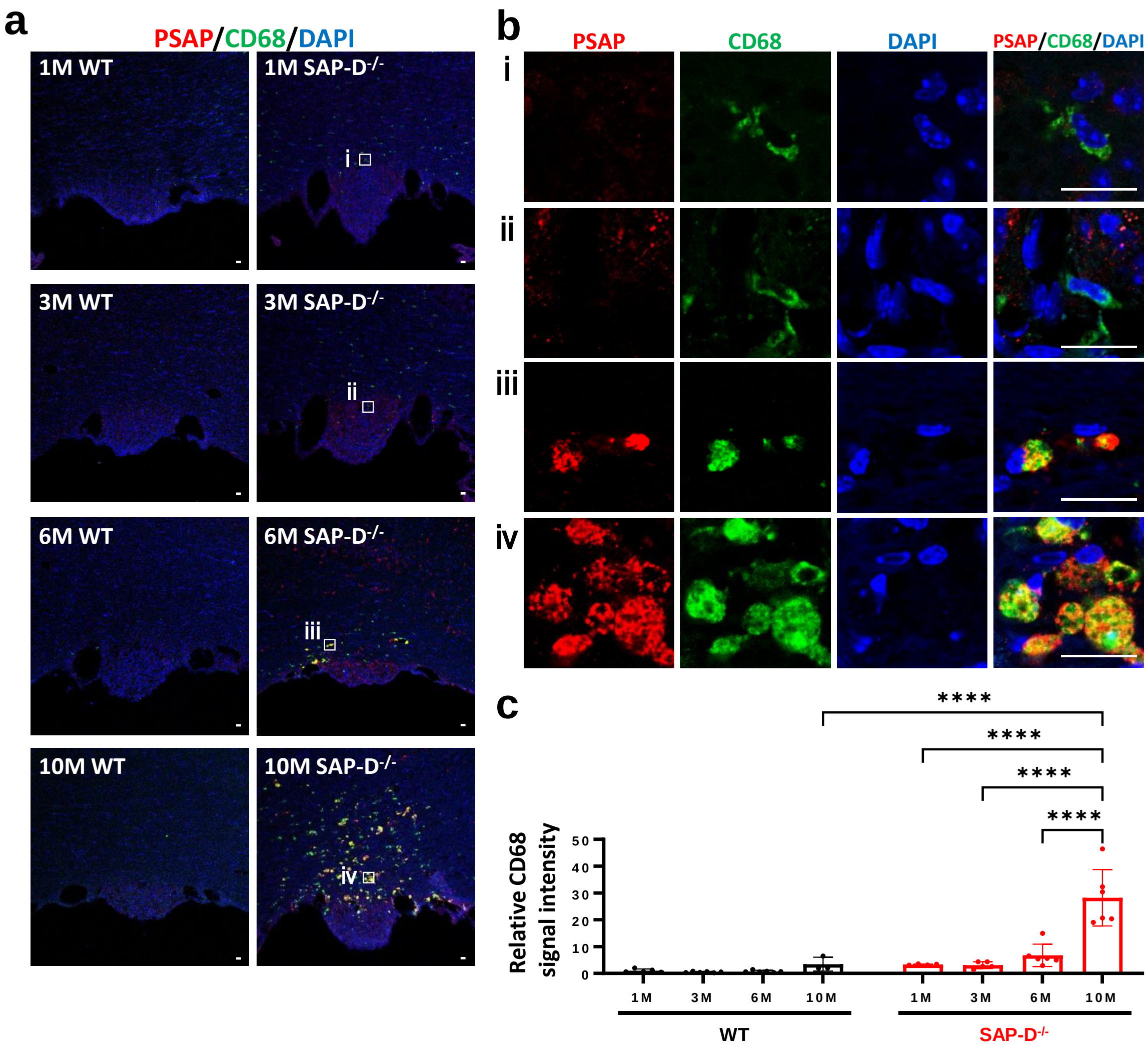

Figure S8. Transformation of microglial morphology in female SAP-D$^{-/-}$ mice

a) Double immunostaining for PSAP (red) and CD68 (green) around the SFO in WT and SAP-D$^{-/-}$ mice at 1, 3, 6, and 10 months of age. b) Enlarged white i–iv squares in (a), as indicated. The nuclei were stained with DAPI (blue). All scale bars indicate 20 µm. c) The relative CD68 signal intensity was calculated by measuring the CD68-positive area and the intensity around the SFO in each section. These values were then expressed relative to the values obtained for the 1-month age group. Two-way ANOVA revealed a significant main effect of age ($F(3,33) = 18.78$, $p < 0.0001$, $\eta p^2 = 0.25$) and genotype ($F(1,33) = 38.05$, $p < 0.0001$, $\eta p^2 = 0.18$), as well as a significant age × genotype interaction ($F(3,33) = 11.98$, $p < 0.0001$, $\eta p^2 = 0.18$). Post-hoc Tukey's tests revealed significant effects between WT-10M and SAP-D$^{-/-}$-10M ($p < 0.0001$, Cohen's $d = 2.74$, 95% CI [−35.53, −13.98]), SAP-D$^{-/-}$-1M and SAP-D$^{-/-}$-10M ($p < 0.0001$, Cohen's $d = 2.98$, 95% CI [−34.35, −15.90]), SAP-D$^{-/-}$-3M and SAP-D$^{-/-}$-10M ($p < 0.0001$, Cohen's $d = 3.17$, 95% CI [−30.26, −12.66]), and SAP-D$^{-/-}$-6M and SAP-D$^{-/-}$-10M ($p < 0.0001$, Cohen's $d = 2.67$, 95% CI [−30.26, −12.66]). **** $p < 0.0001$.

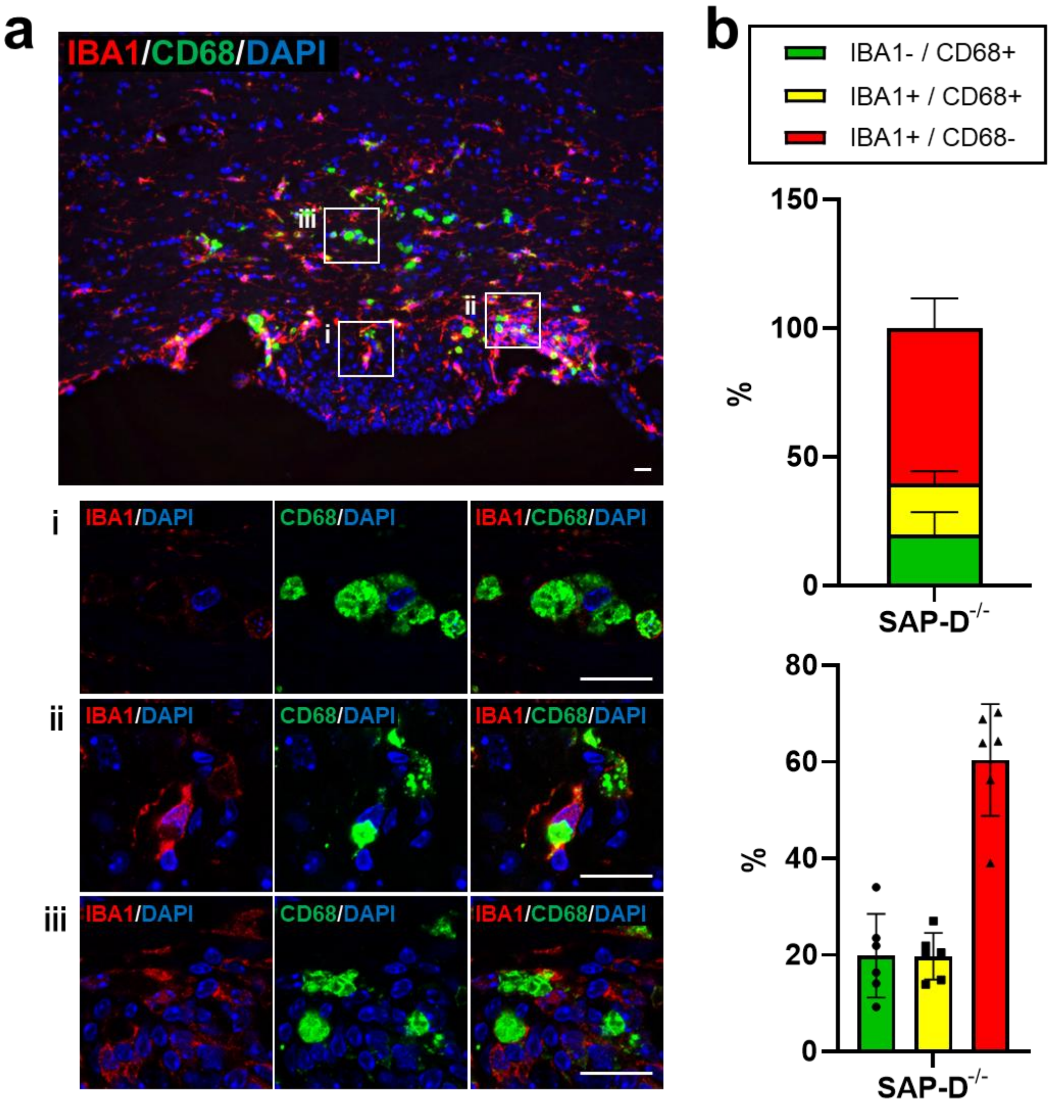

Figure S9. Co-localization of IBA1 and CD68 in SFO and surrounding tissues

a) Double immunofluorescence staining of IBA1 (red) and CD68 (green) around the SFO in 10-month-old female SAP-D$^{-/-}$ mice. White squares are magnified in i–iii. Nuclei are shown with DAPI (blue) staining. All scale bars = 20 mm. b) Areas of co-localization of CD68 and IBA1 in the SFO and surrounding tissues of SAP-D$^{-/-}$ mice were quantified (%). The upper panel presents a stacked bar chart, whereas the lower panel shows the individual data values using a bar chart format. Data are shown as the mean ± SD (n = 6).

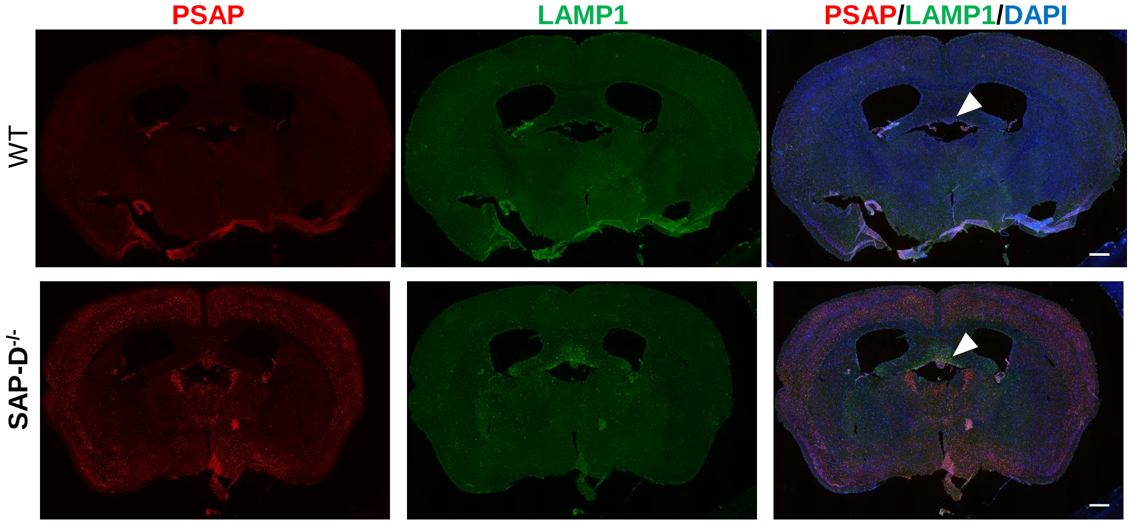

Figure S10. Increased LAMP1 staining around the SFO in 10-month-old female mice

Immunostaining of PSAP (red) and LAMP1 (green) in coronal brain sections containing the SFO in 10-month-old female WT and SAP-D$^{-/-}$ mice. White arrowheads indicate SFO regions. Nuclei were stained with DAPI (blue). Scale bars, 500 µm.

a

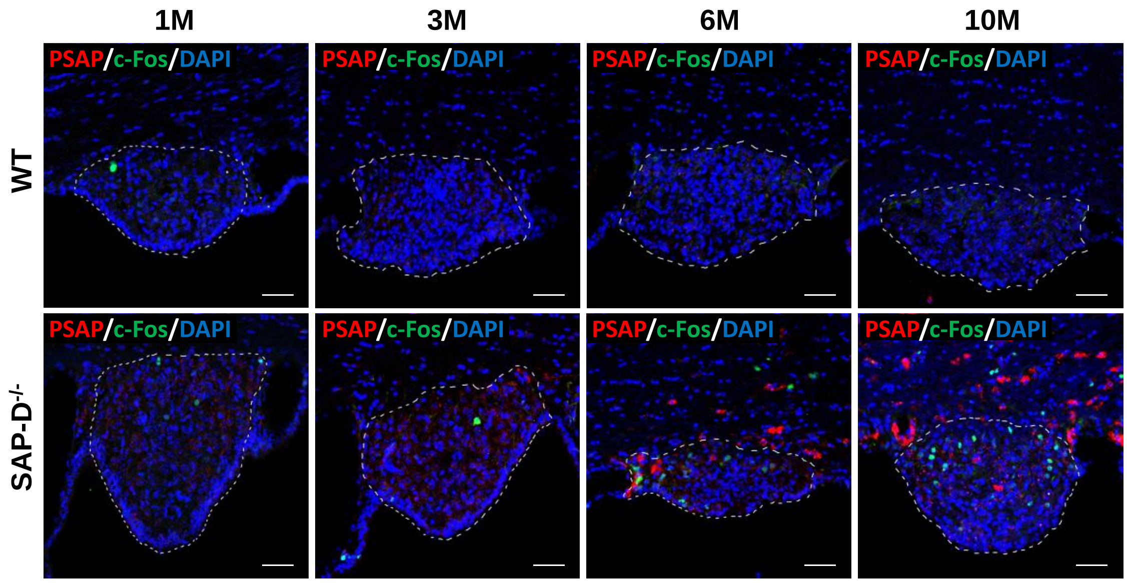

b

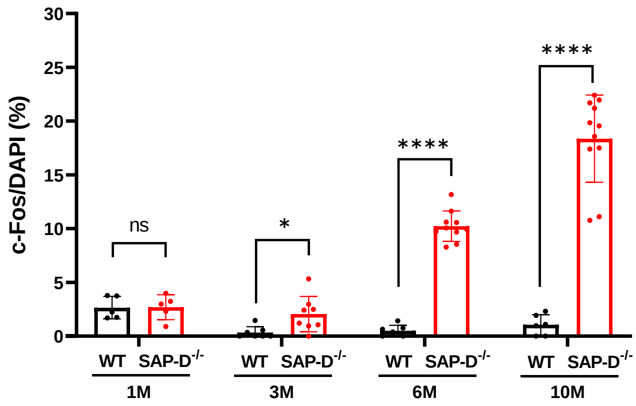

Figure S11. Increased c-Fos expression with age in SAP-D$^{-/-}$ female mice

a) Double immunofluorescence staining of PSAP (red) and c-Fos (green) in the SFO of 1-, 3-, 6-, and 10-month-old female WT and SAP-D$^{-/-}$ mice. The white dotted lines enclose the SFO. The nuclei were counterstained with DAPI (blue). Scale bar: 50 μm. b) Quantification of c-Fos-positive cell ratios in the SFO from (a). Data represent the mean ± SD of 5–11 sections per group, with individual section values shown as dots. The results of Student's t-tests comparing WT and SAP-D$^{-/-}$ mice at each age are as follows: 1M, p = 0.9496, Cohen's d = 0.04 (95% CI: −1.56, 1.65); 3M, p = 0.0209, Cohen's d = 1.36 (95% CI: 0.30, 3.12); 6M, p < 0.0001, Cohen's d = 8.50 (95% CI: 8.51, 10.91); and 10M, p < 0.0001, Cohen's d = 5.16 (95% CI: 13.68, 20.93). **** p < 0.0001, * p < 0.05. ns: no significant difference.

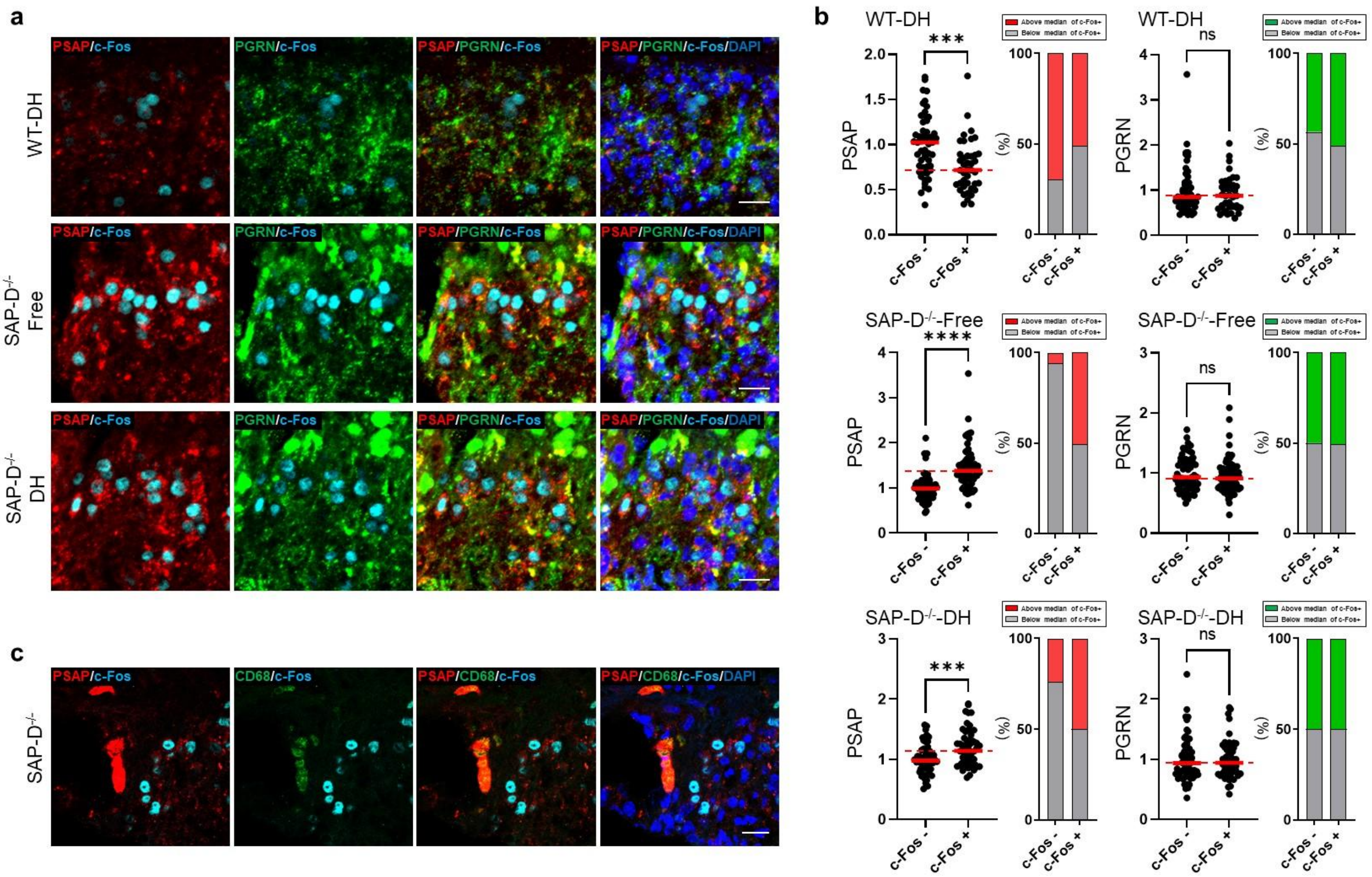

Figure S12. c-Fos expressing cells in the SFO in SAP-D$^{-/-}$ mice tended to accumulate PSAP

a) Triple immunofluorescent staining of PSAP (red), PGRN (green), and CD68 (cyan) in the SFO of 10-month-old female WT and SAP-D$^{-/-}$ mice. WT mice were treated with 24 h water deprivation (indicated as DH) to induce the DH-SAP-D$^{-/-}$ mice were treated with free access to drinking water (indicated as Free) or DH. **b)** Quantification of PSAP and PGRN staining intensity in the c-Fos-positive or -negative cells in **a**. The median for each group is indicated by a red line. The results of Student's t-tests for each panel are as follows: PSAP-WT-DH, p = 0.0003, Cohen's d = 0.78 (95% CI: −0.37, −0.11); PGRN-WT-DH, p = 0.4088, Cohen's d = 0.17 (95% CI: −0.27, 0.11); PSAP-SAP-D$^{-/-}$-Free, p < 0.0001, Cohen's d = 1.14 (95% CI: 0.30, 0.56); PGRN-SAP-D$^{-/-}$-Free, p = 0.2564, Cohen's d = 0.19 (95% CI: −0.15, 0.04); PSAP-SAP-D$^{-/-}$-DH, p = 0.0006, Cohen's d = 0.68 (95% CI: 0.08, 0.29); and PGRN-SAP-D$^{-/-}$-DH, p = 0.9673, Cohen's d = 0.007 (95% CI: −0.13, 0.13). The median in each c-Fos positive group was used as the threshold (indicated by the red dotted lines), and the percentages of values above and below the median are also presented. Circles indicate each cell. Number of cells in each group: WT-DH-c-Fos - (N = 53), WT-DH-c-Fos + (N = 41), SAP-D$^{-/-}$-Free-c-Fos - (N = 66), SAP-D$^{-/-}$-Free-c-Fos + (N = 71), SAP-D$^{-/-}$-DH-c-Fos - (N = 54), and SAP-D$^{-/-}$-DH-c-Fos + (N = 54). ns: no

significant difference. *** $p < 0.001$, **** $p < 0.0001$. **c)** Triple immunofluorescent staining of PSAP (red), CD68 (green), and c-Fos (cyan) in the SFO of 10-month-old female SAP-$D^{-/-}$ mice was presented to show that the PSAP and c-Fos co-expressing cells were negative for the CD68 immuno-staining. Nuclei are labeled by DAPI (blue) staining. All scale bars, 20 μm.

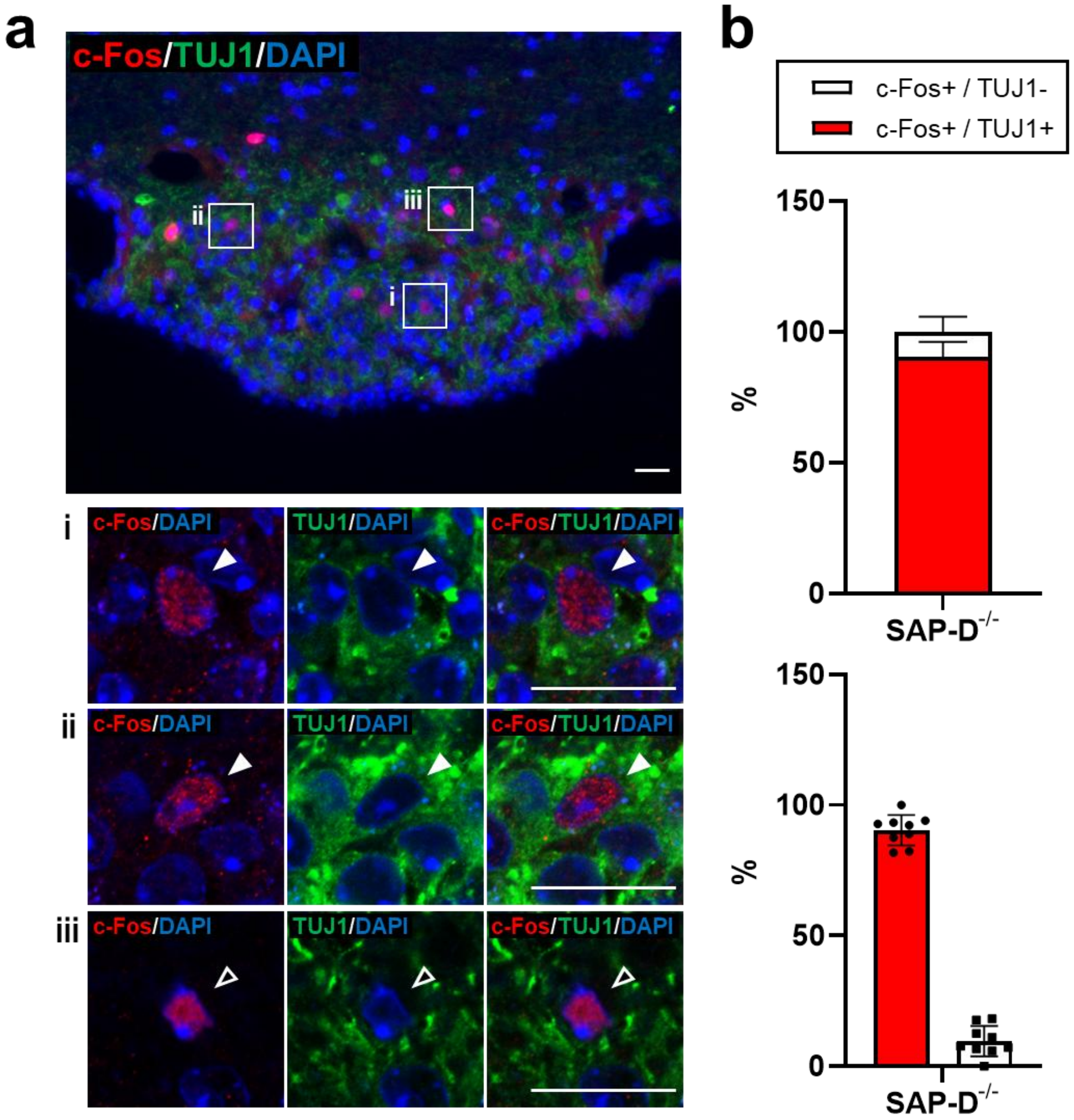

Figure S13. Co-localization of c-Fos and TUJ1 in SFO.

a) Double immunofluorescence staining of c-Fos (red) and TUJ1 (green) around the SFO in 10-month-old female SAP-D$^{-/-}$ mice. TUJ1 antibody (ab14545, Abcam, Cambridge, UK) was used as a neuron-specific marker. white squares are magnified in i–iii. White arrowheads indicate TUJ1-positive neurons exhibiting c-Fos expression (i and ii). Open arrowheads indicate TUJ1-negative cells exhibiting c-Fos expression (iii). Nuclei are labeled with DAPI (blue) staining. All scale bars = 20 μm. b) Co-localization of c-Fos and TUJ1 in the SFO of SAP-D$^{-/-}$ mice was quantified (%). The upper panel presents a stacked bar chart, whereas the lower figure shows the individual data values using a bar chart format. Data are shown as the mean ± SD (n = 9).

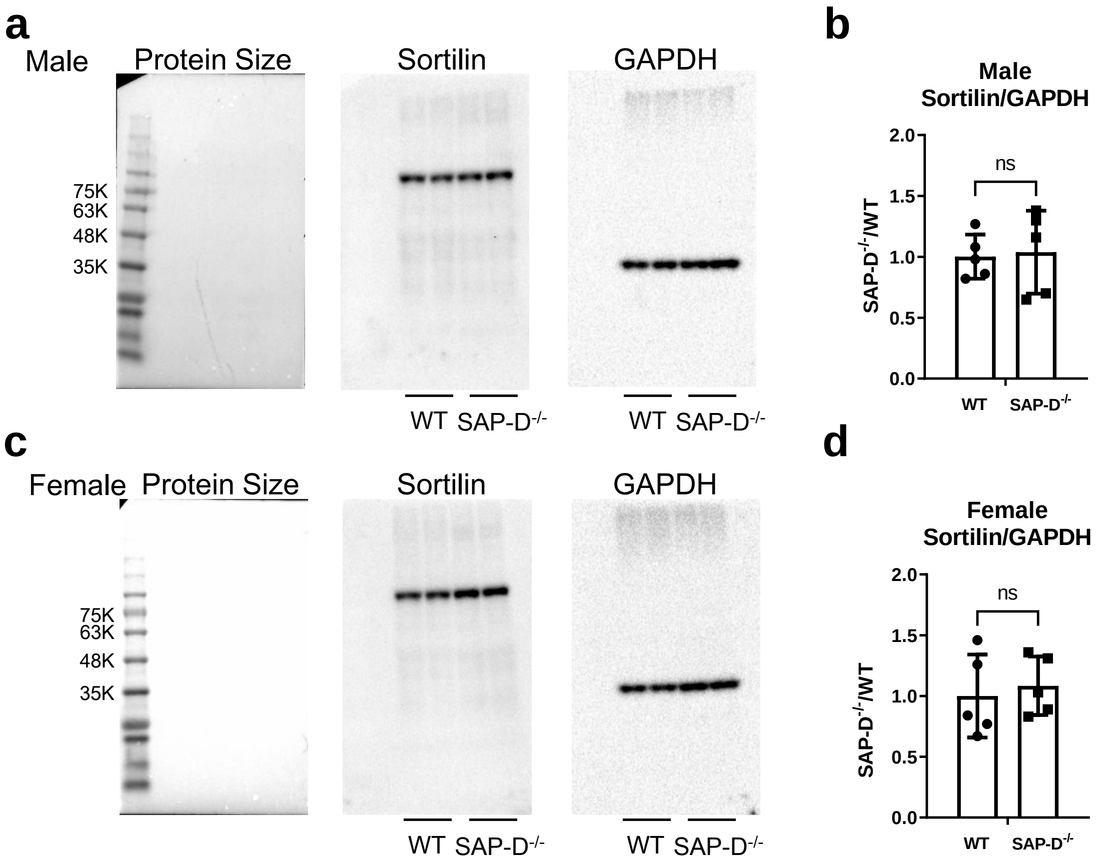

Figure S14. Sortilin expression in the SFO of 10-month-old mice

Western blot analysis of sortilin (12369-1-AP, Proteintech, USA) in the SFO of 10-month-old male (a) and female (c) mice. Samples were obtained as shown in Figure 1c. Western blot quantification via densitometric analysis (b and d). Expression levels were normalized to GAPDH and presented as a ratio relative to WT. The results of Student's t-tests for (b) and (d) are as follows: male sortilin (b), $p = 0.8401$, Cohen's $d = 0.13$ (95% CI: −0.36, 0.43); and female sortilin (d), $p = 0.6651$, Cohen's $d = 0.28$ (95% CI: −0.34, 0.51). Bars indicate the mean ± SD of 5 mice per group. Statistical analysis was performed using a t-test.

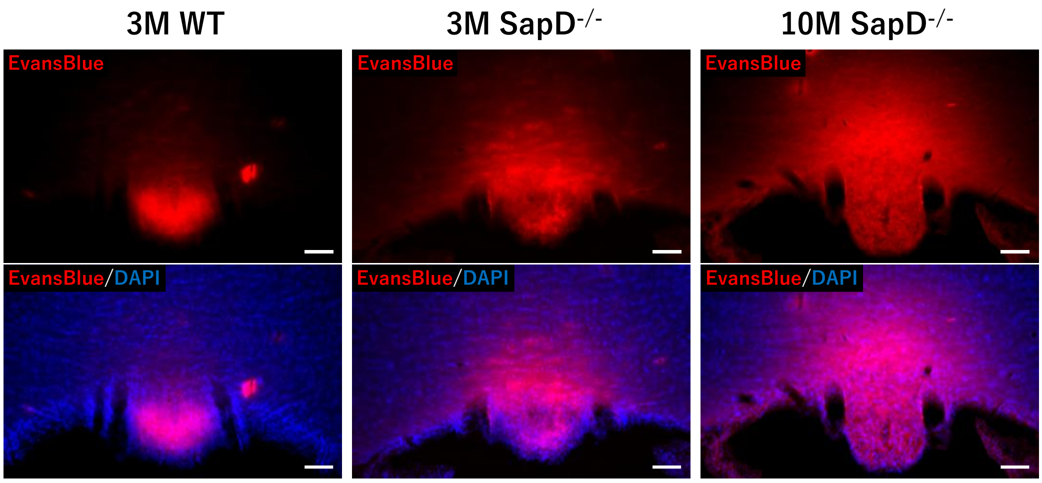

Figure S15. Analysis of blood–brain barrier integrity in the SFO region using Evans blue permeability assay

To assess blood–brain barrier integrity in the SFO and surrounding fornix of WT and SAP-D$^{-/-}$ mice, Evans blue administration experiments were performed. The mice were given a tail vein injection of 2% (w/v) Evans blue dissolved in saline (4 mL/kg body weight). Thirty minutes after injection, mice were anesthetized with isoflurane and reflux-fixed in 4% paraformaldehyde in 50 mM phosphate-buffer (PFA), followed by additional refluxing with PBS. The brains were extracted and immersion-fixed in 4% PFA for 48 h, followed by substitution with 30% sucrose in PBS and embedding in Tissue Tek OCT (Sakura Finetek USA, CA, USA) for cryopreservation. Next, 30-μm frozen coronal sections containing the SFO were obtained using a Leica CM3050 S cryostat (Leica Microsystems GmbH, Wetzlar, Germany). The sections were permeabilized with PBS-T (0.4% Triton-X) for 24 h at room temperature and mounted using Vectashield Mounting Medium containing 4′,6-diamidino-2-phenylindole (DAPI) (Vector, Burlingame, CA, USA). Evans blue fluorescence (red) was visualized under a BZ-X800 microscope (KEYENCE, Osaka, Japan) using a Cy3 fluorescent filter, with DAPI-stained nuclei (blue). Evans blue binds to albumin and selectively permeates capillaries in brain regions lacking an intact blood-brain barrier. In 3-month-old WT female mice, Evans blue permeability was restricted to the SFO; the surrounding fornix remained unstained, confirming the absence of a blood–brain barrier within the SFO and the presence of an intact blood-brain barrier in the surrounding fornix region. In contrast, in 3-month-old SAP-D$^{-/-}$ female mice, Evans blue signal was detected in the SFO as well as in the surrounding fornix, indicating disruption of the functional blood-brain barrier in the surrounding fornix. This tendency was observed to further progress at 10 months of age. Scale bar: 50 μm.

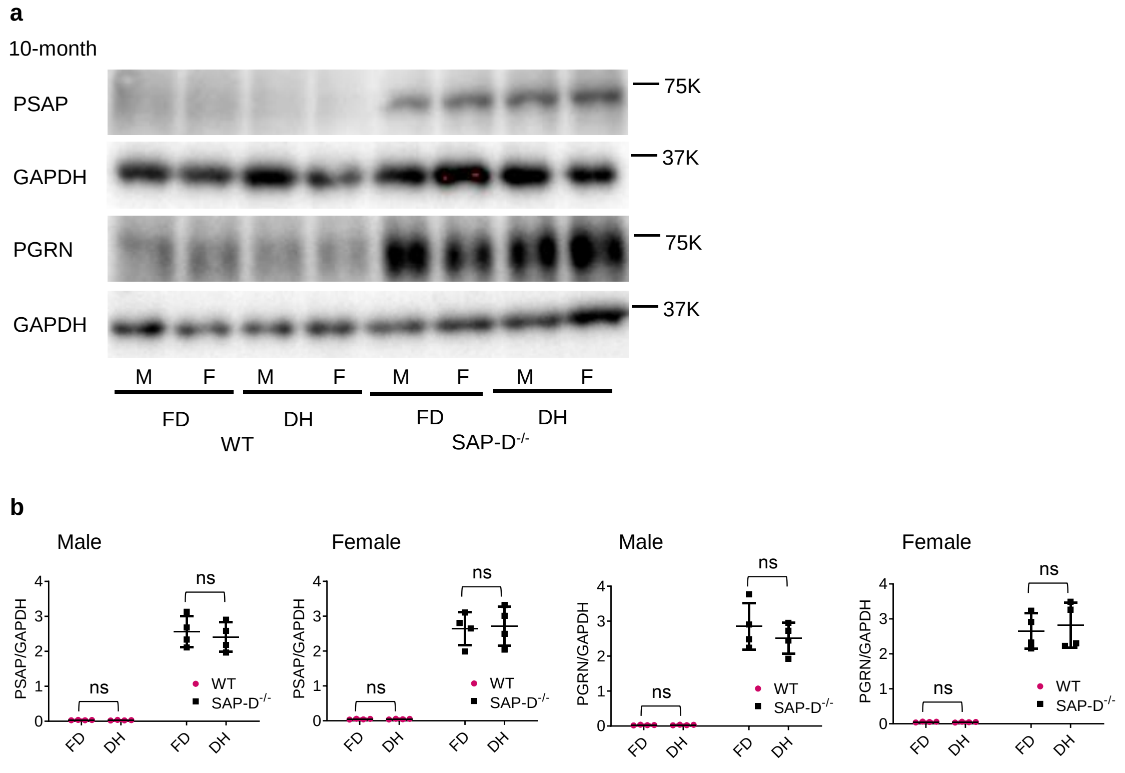

Figure S16. Effect of dehydration on PSAP and PGRN protein expression in the SFO of WT and SAP-D$^{-/-}$ mice

a) Western blotting for PSAP, PGRN, and GAPDH in the SFO. Mice were dehydrated for 24 h (DH) or allowed to freely consume water (FD). The cerebral region containing the SFO (0.7–0.8 mm posterior to bregma) was used for protein extraction and immunoblotting. M, male; F, female. b) Quantification of the data in (a). Data are presented as the mean ± SD (n = 4). Each point indicates an individual value in each group. Statistical analysis was performed using a Student's t-test. ns: not significant. The results of Student's t-tests for each panel are as follows: Male-PSAP/GAPDH-WT, $p = 0.4536$, Cohen's $d = 0.56$ (95% CI: −0.014, 0.007); Male-PSAP/GAPDH-SAP-D$^{-/-}$, $p = 0.6264$, Cohen's $d = 0.36$ (95% CI: −0.90, 0.59); Female-PSAP/GAPDH-WT, $p = 0.9473$, Cohen's $d = 0.04$ (95% CI: −0.017, 0.018); Female-PSAP/GAPDH-SAP-D$^{-/-}$, $p = 0.8349$, Cohen's $d = 0.15$ (95% CI: −0.81, 0.97); Male-PGRN/GAPDH-WT, $p = 0.7531$, Cohen's $d = 0.23$ (95% CI: −0.011, 0.014); Male-PGRN/GAPDH-SAP-D$^{-/-}$, $p = 0.4303$, Cohen's $d = 0.59$ (95% CI: −1.32, 0.64); Female-PGRN/GAPDH-WT, $p = 0.4745$, Cohen's $d = 0.53$ (95% CI: −0.024, 0.012); and Female-PGRN/GAPDH-SAP-D$^{-/-}$, $p = 0.7010$, Cohen's $d = 0.28$ (95% CI: −0.83, 1.17).

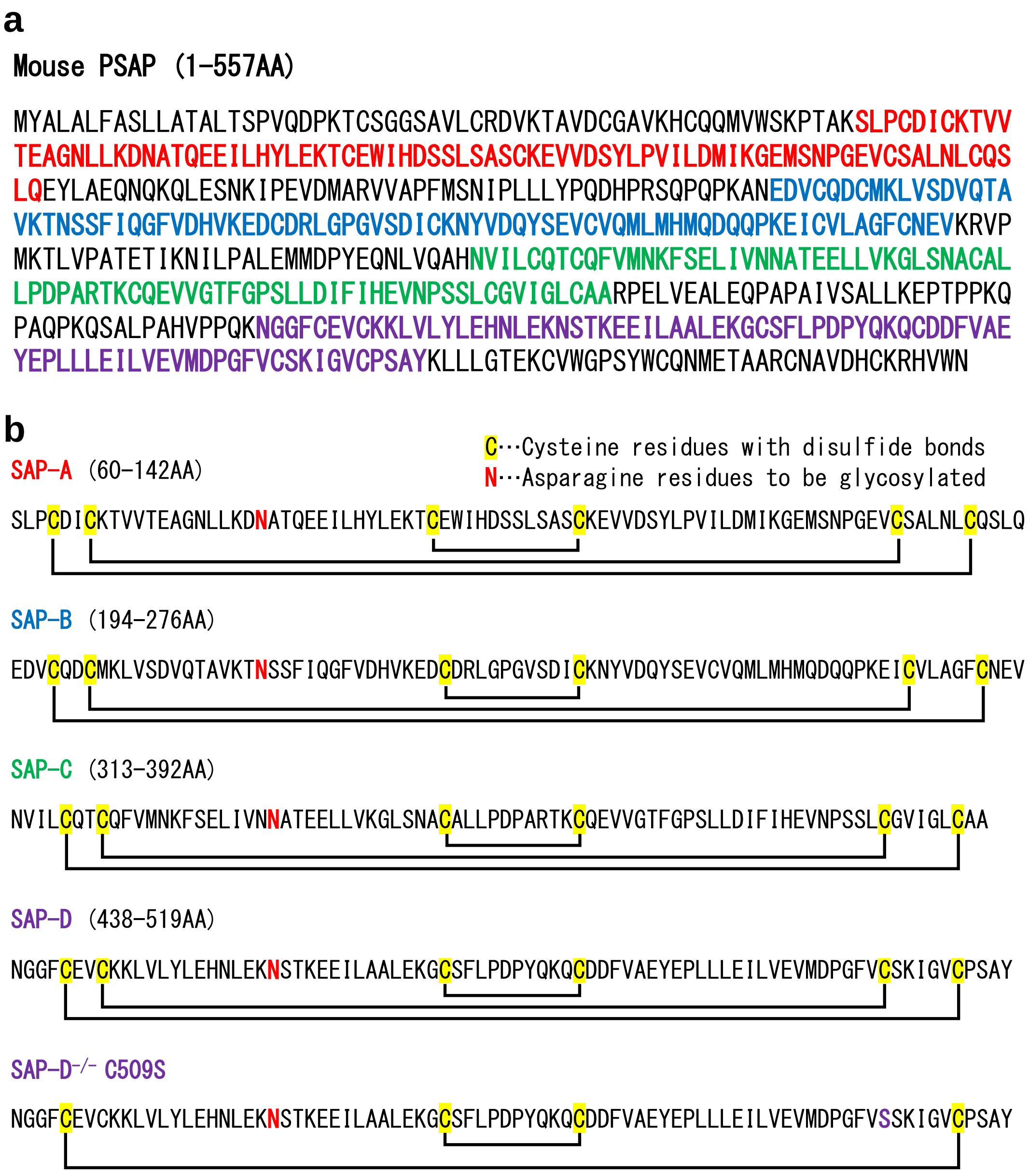

Figure S17. Amino acid (AA) sequence of mouse PSAP and the respective structures of SAP-A–D produced from PSAP

a) AA sequence of mouse PSAP (AAs 1–557). The respective corresponding AAs are presented in red for SAP-A, blue for SAP-B, green for SAP-C, and purple for SAP-D. b) The AA sequences of SAP-A (AAs 60–1442), SAP-B (AAs 194–276), SAP-C (AAs 313–392), and SAP-D (AAs 438–519) are presented. Cysteine residues that form disulfide bonds and asparagine residues that can be glycosylated are indicated. The AA sequence of SAP-D$^{-/-}$ (C509S) used in this study is presented at the bottom.

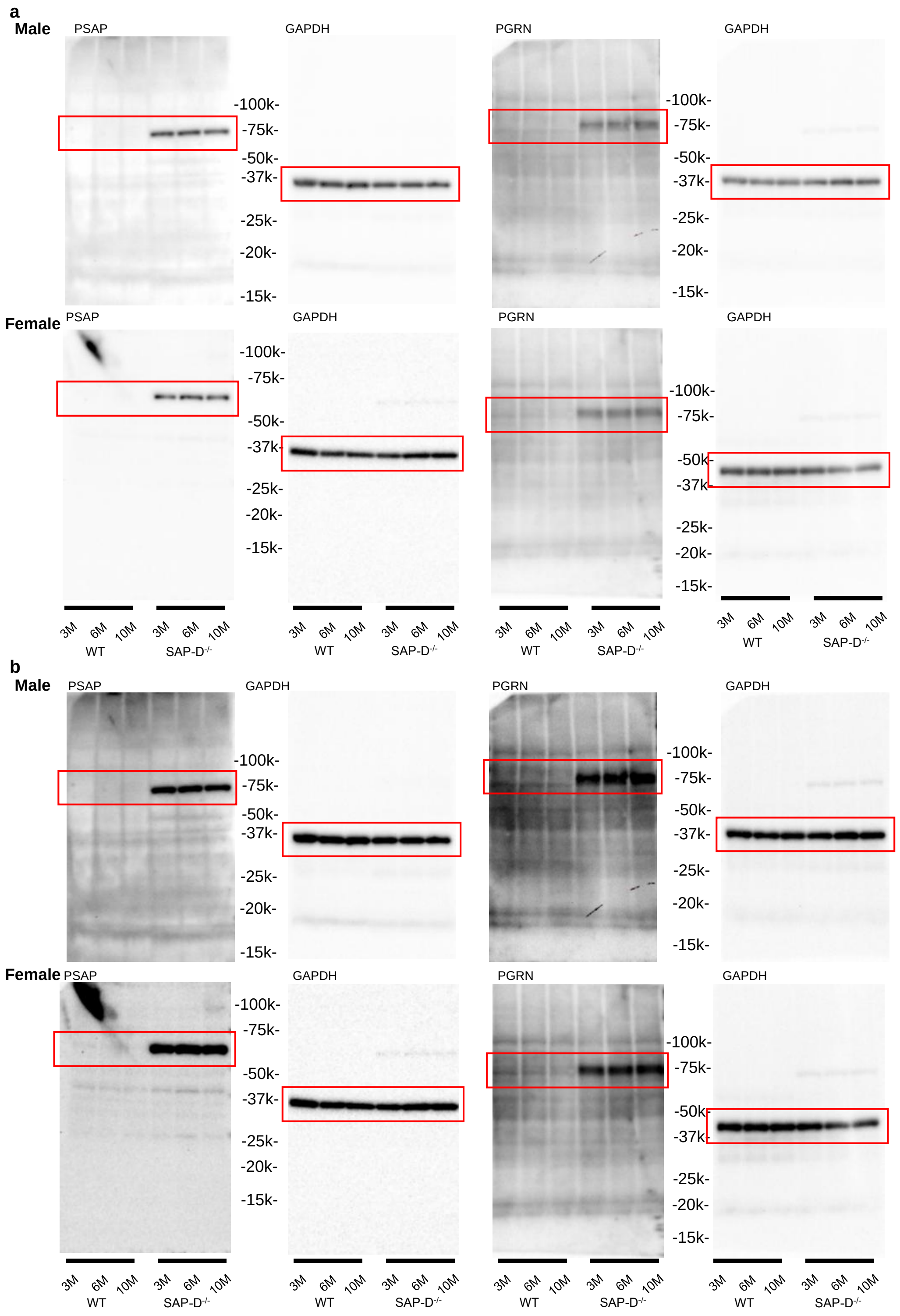

Figure S18. **Full-scale western blot of Figure 1d**

Uncropped blots used in Figure 1d. Short exposure (a) and long exposure (b) of the same immunoblots.

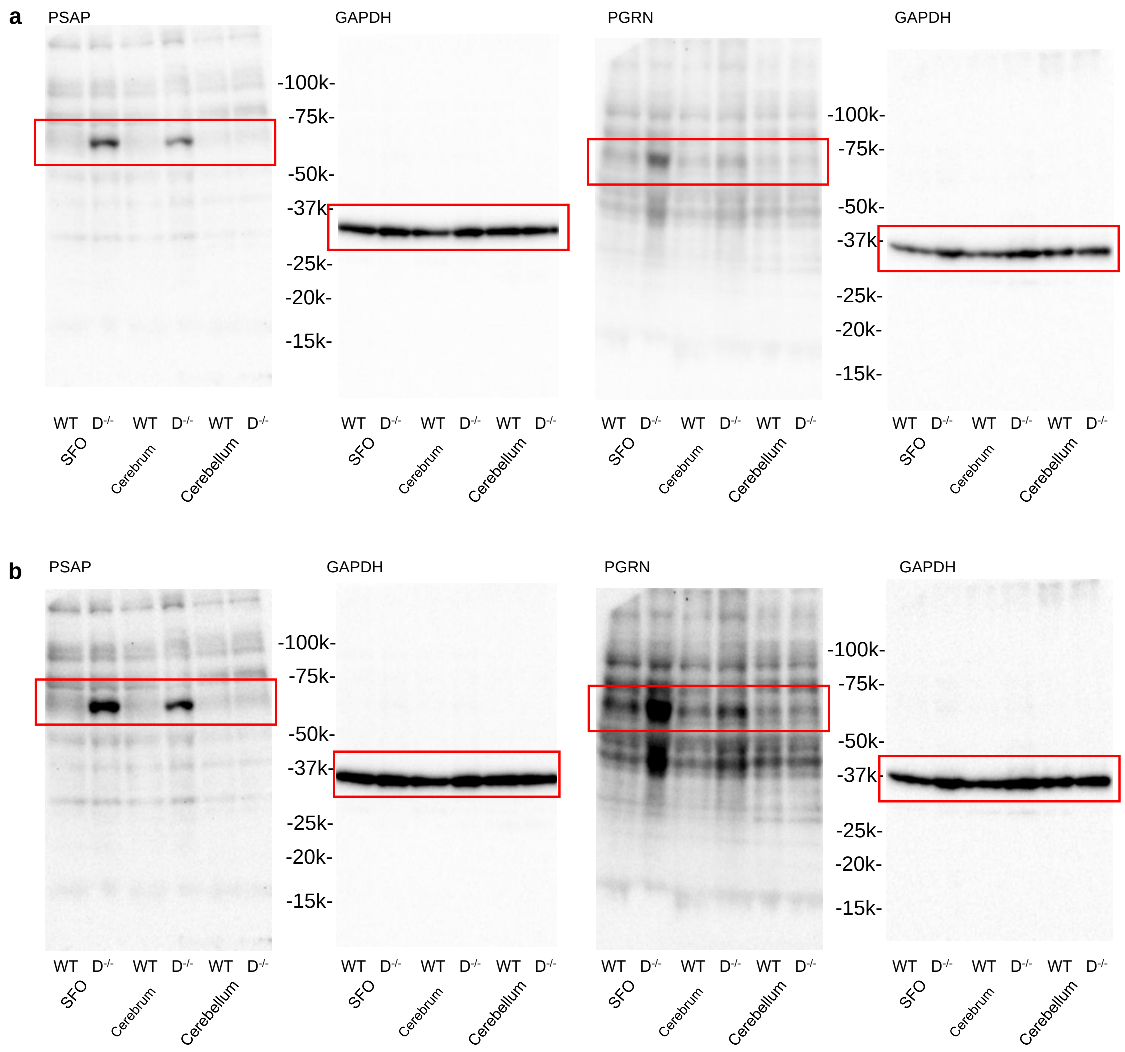

Figure S19. Full-scale western blot of Figure 1g

Uncropped blots are shown Figure 1g. Short exposure (a) and long exposure (b) of the same immunoblots.

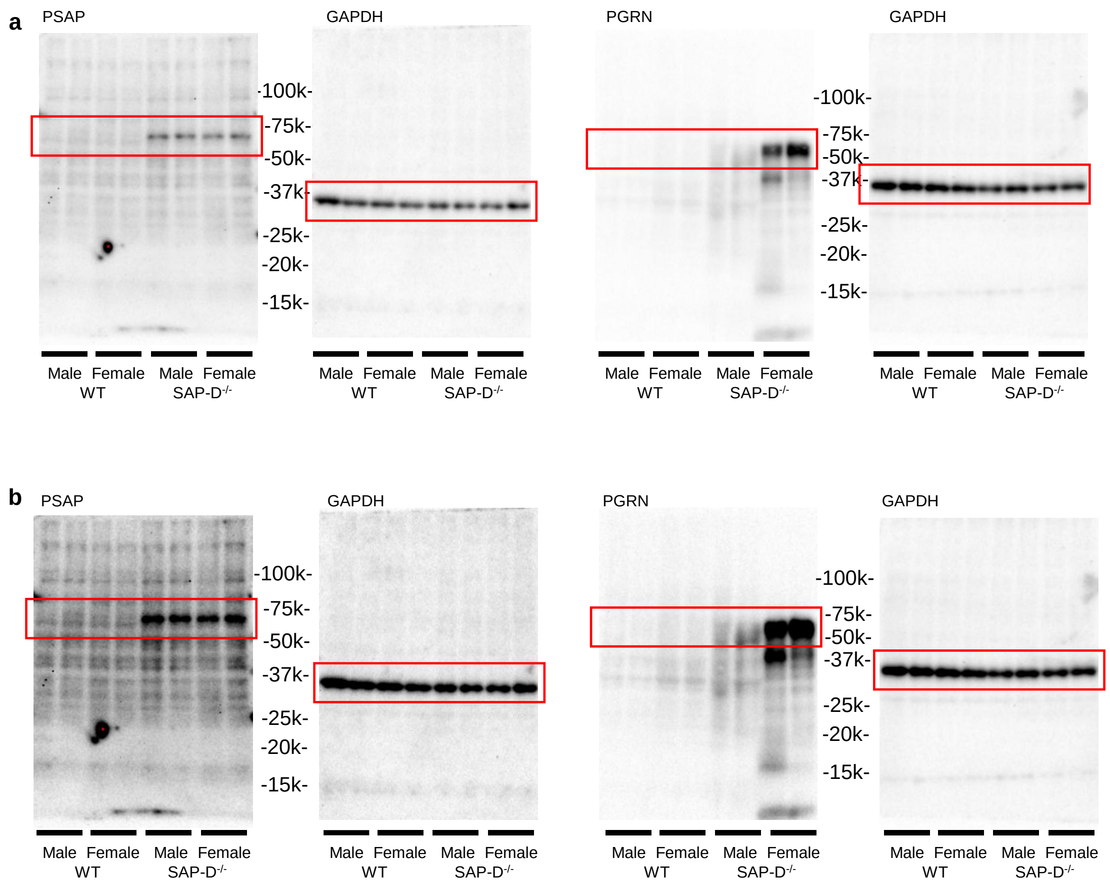

Figure S20. Full-scale western blotting for Supplemental Figure S4a

Uncropped blots used in Supplemental Figure S4a. Short exposure (a) and long exposure (b) of the same immunoblots.

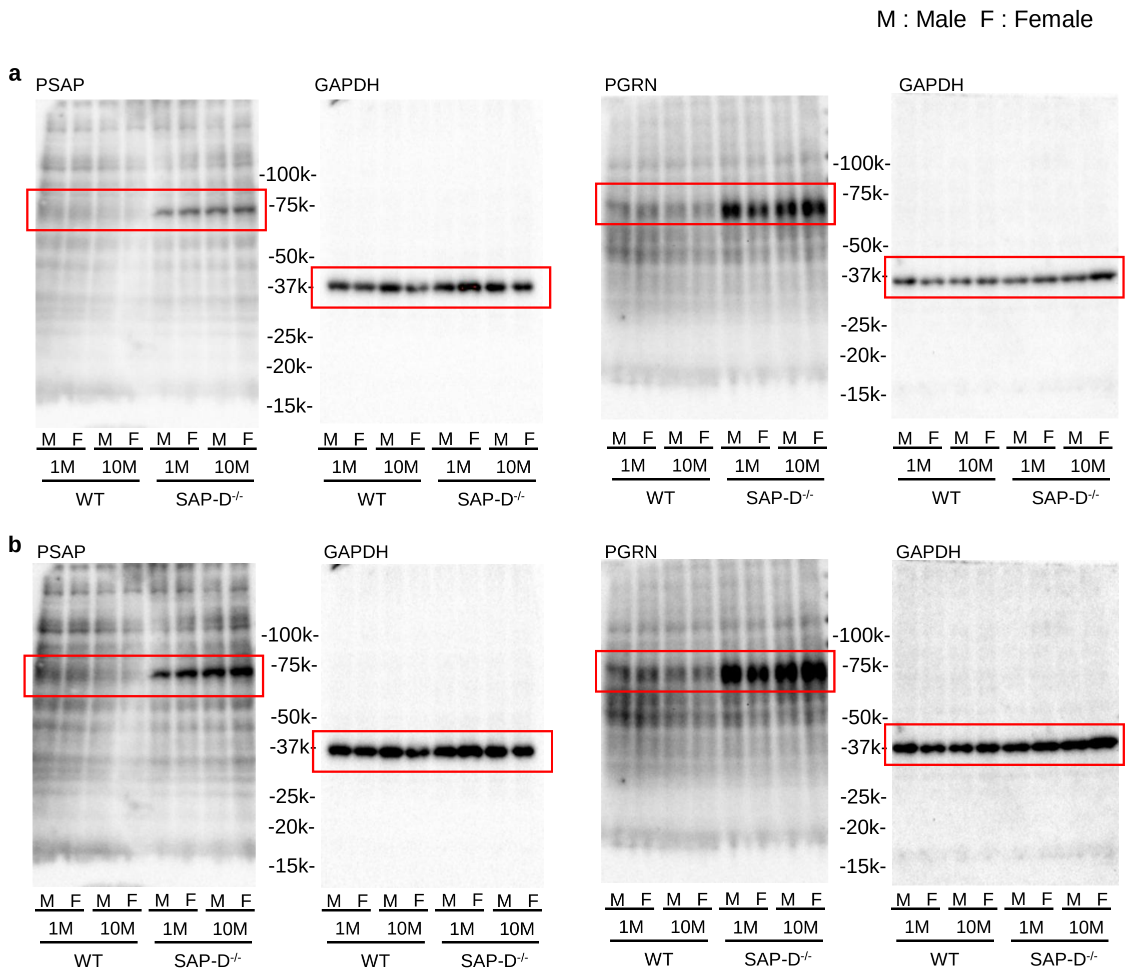

Figure S21. Full-scale western blot analysis of Supplemental Figure S16a

Uncropped blots used in Supplemental Figure S16a. Short exposure (a) and long exposure (b) of the same immunoblots.
